# An immunomodulating peptide to counteract solar radiation-induced immunosuppression and DNA damage

**DOI:** 10.1038/s41598-023-38890-4

**Published:** 2023-07-20

**Authors:** Michael Agrez, Mark Stephen Rybchyn, Warusavithana Gunawardena Manori De Silva, Rebecca Sara Mason, Christopher Chandler, Terrence J. Piva, Kristofer Thurecht, Nicholas Fletcher, Feifei Liu, Gayathri Subramaniam, Christopher B. Howard, Benjamin Blyth, Stephen Parker, Darryl Turner, Justyna Rzepecka, Gavin Knox, Anastasia Nika, Andrew Hall, Hayley Gooding, Laura Gallagher

**Affiliations:** 1 InterK Peptide Therapeutics Limited, Sydney, NSW Australia; 2 School of Medical Sciences and Bosch Institute, University of Sydney, Sydney, Australia; 3 Charles Perkins Centre and School of Life and Environmental Sciences, University of Sydney, Sydney, Australia; 4 Auspep Pty Limited, Melbourne, Australia; 5 Health and Biomedical Sciences, RMIT University, Melbourne, Australia; 6 Centre for Advanced Imaging, University of Queensland, Brisbane, Australia; 7 Australian Institute for Bioengineering and Nanotechnology and the ARC Training Centre for Innovation in Biomedical Imaging Technologies, University of Queensland, Brisbane, Australia; 8 Peter MacCallum Cancer Centre and Sir Peter MacCallum Department of Oncology at the University of Melbourne, Melbourne, Australia; 9 Concept Life Sciences Limited, Edinburgh, Scotland

**Keywords:** T cells, CD4-positive T cells, CD8-positive T cells, Interferons, Interleukins, Basal cell carcinoma, Melanoma, Squamous cell carcinoma, Senescence, DNA damage response, Cancer prevention, Telomeres, Tumour immunology

## Abstract

Ultraviolet radiation (UVR) induces immunosuppression and DNA damage, both of which contribute to the rising global incidence of skin cancer including melanoma. Nucleotide excision repair, which is activated upon UVR-induced DNA damage, is linked to expression of interleukin-12 (IL-12) which serves to limit immunosuppression and augment the DNA repair process. Herein, we report an immunomodulating peptide, designated IK14800, that not only elicits secretion of IL-12, interleukin-2 (IL-2) and interferon-gamma (IFN-γ) but also reduces DNA damage in the skin following exposure to UVR. Combined with re-invigoration of exhausted CD4+ T cells, inhibition of UVR-induced MMP-1 release and suppression of B16F10 melanoma metastases, IK14800 offers an opportunity to gain further insight into mechanisms underlying the development and progression of skin cancers.

## Introduction

Immunosuppression is associated with exposure to ultraviolet radiation (UVR)^1–3^, ageing^4^ and manifests within a tumour micro-environment (TME) as T cell exhaustion^5^. UVR-induced DNA damage and immunosuppression are recognised as major risk factors in the development of keratinocyte cancers and melanoma^6,7^. Both UVA^8^ and UVB^4^ spectra cause DNA damage that is linked to immunosuppression^9,10^. Skin cancers also exhibit elevated expression of the Src family kinase member, c-Src^11^. Notably, activation of c-Src upon UVR exposure is well-recognised^12–14^ and we have previously reported that a 10 mer peptide, RSKAKNPLYR, linked to a hydrophobic signal peptide, inhibits c-Src activity and proliferation of cancer cells *in vitro*^15^.

Exposure to UVR results in the formation of cyclobutane pyrimidine dimers (CPDs) and oxidative DNA damage in the form of 8-oxo-7,8-dihydro-2’-deoxyguanosine (8-OHdG) which can be prevented by the vitamin D hormone, 1,25 Dihydroxyvitamin D_3_ and related compounds^16^. Furthermore, if a cell is so severely damaged by UVR that it cannot remove DNA lesions, apoptosis is induced which eliminates that cell and apoptotic keratinocytes (sunburn cells) are frequently found in UVR-exposed epidermis^17^. While CPDs are substrates of nucleotide excision repair (NER), 8-OHdG is subject to base excision repair rather than NER and recent evidence suggests that NER, which reduces UVR-mediated DNA damage, can be augmented in the presence of interleukin-12 (IL-12) which provides a link between DNA repair and prevention of UVR-induced immunosuppression^17–20^. Furthermore, there is no major effect of IL-12 on transcription of apoptosis-related proteins^21^ and because UV-induced apoptosis is a result of DNA damage^17^, it has been suggested that IL-12-mediated inhibition of apoptosis may be an indirect effect of the DNA repair pathway^21^. This hypothesis is supported by the finding that apoptosis is reduced in the skin of wild-type UVR-exposed mice but not in mice lacking the xeroderma pigmentosum gene, i.e., *XPA* involved in NER^17^. However, IL-12 is not itself a DNA repair protein in the UV-induced NER pathway. Rather, it appears that DNA damage-sensing proteins bind to sites of DNA damage with recruitment of repair machinery to the site and that dysregulation of this pathway is involved in carcinogenesis^22^.

A deficiency in Th1 cytokines upon UVB exposure^23^ is also observed in the immune profiles of sera from melanoma patients which display marked reduction in levels of IFN-γ and IL-2^24,25^. IL-12 is comprised of two subunits, p35 and p40, which need to be co-expressed in the same cell to secrete bioactive IL-12p70^26^. The cytokine is expressed in dendritic cells (DCs) including Langerhans cells (LCs) in the skin, keratinocytes^27^ and has also been detected in T cells^28,29^. Notably, exposure of skin to UVR results in depletion of epidermal LCs^30^ with potential, thereby, to lessen tissue IL-12 levels. Production of IL-12 by mature DCs regulates IFN-y production by pro-inflammatory Th1-differentiated T cells^31^ and IL-12 can synergise with either IL-2 or IL-18 to promote IFN-γ production which acts on antigen-presenting cells (APCs) to increase IL-12 secretion in a positive-feedback loop^32^. Hence, in murine models, IL-12 counteracts UVB-induced immunosuppression^33,34^. However, UVB suppresses IFN-γ production^35,36^ in association with expansion of immunosuppressive CD4+ T regulatory (Treg) cells^37^ and IL-12-deficient mice display enhanced photo-carcinogenesis^38^. These observations are consistent with reported antitumour effects for IL-12 in preclinical models of cancer such as B16 murine melanoma and in melanoma patients^26,39^.

In contrast to IL-12, IL-2 is produced primarily by activated CD4+ T cells^40^. Mononuclear cells from older individuals and aged mice produce less IL-2 and IFN-γ^41^ which is also seen with T cell exhaustion^42^. Costimulatory receptors and their respective ligands on T cells and DCs play an important role in both IL-2 and IL-12 production consequent upon T cell-APC engagement. For example, expression of the ligand for the CD40 receptor, i.e., CD40L, in activated T cells is critical for IL-12 production by APCs^43^ and much greater amounts of IL-12 are produced when DCs are stimulated by ligation of their CD40 receptor^44^ with CD4+ T cell-expressed CD40L^31,45^. In addition, the quintessential costimulatory receptor expressed on T cells, CD28, is required for full T cell activation and IL-2 production which promotes cell proliferation^46^. Loss of CD28 expression is a hallmark of the age-associated decline of CD4+ T cell function^46,47^ and CD4+ CD28 null T cells can comprise up to half of the total CD4+ T cell compartment in some older individuals^48^. Moreover, CD28 re-enforces the positive feedback loop between IL-2 and its high-affinity receptor subunit, CD25^49^, which ensures an optimal response to TCR stimulation^50,51^. Importantly, IL-12 can rescue senescent CD4+ CD28 null T cells in combination with anti-CD3 stimulation resulting in re-expression of functional CD28 in a proportion of CD4+ T cells^52^.

The mechanisms underlying UVR-induced immunosuppression and DNA damage that contribute to development of skin cancers remain poorly understood. In the current study we present Th1-skewed cytokine responses induced by a novel peptide, designated IK14800, that comprises the sequence RSKAKNPLYR linked to an octa-arginine (R8) cell-penetrating peptide^53^. In so doing, the potential relevance of this compound to DNA damage reduction including immune dysfunction associated with ageing and T cell exhaustion is highlighted.

## Results

### IK14800 induces IL-2 production and expression of the costimulatory receptor, CD28, in T cells

**Figure 1:**
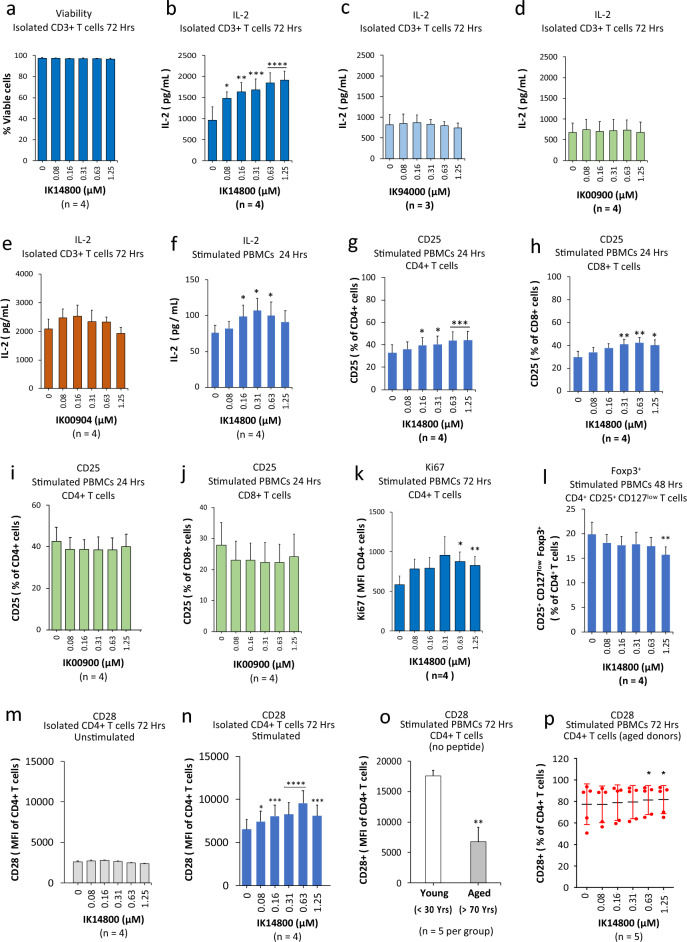
IK14800 induces IL-2 production and expression of the costimulatory receptor, CD28, in T cells. Buffy coat samples were obtained from human volunteers following ethics approval. PBMC and isolated CD3+ T cell cultures were stimulated with either anti-CD3 antibody alone or anti-CD3/anti-CD28 antibodies, respectively, as described in the methods. Flow cytometry was performed on cells cultured for 24–72 h in the presence of either IK14800 (RSKAKNPLYRRRRRRRRR) or IK00900 (RRRRRRRRR) and IL-2 assessed by ELISA in the presence of either IK14800, IK00900, IK94000 (RSKAKNPLY) or the lipidic peptide, IK00904 (RRRRRRRRR linked to 4 dodecanoic acid moieties). Each experiment was performed using triplicate wells (technical replicates) and repeated at least 3 times (n = experimental replicates) as indicated below each panel. All error bars represent standard error of the mean (SEM). Flow cytometry data are shown as mean fluorescence intensity (MFI) and percentage values as indicated in the panels. Dot plots and gating strategies are shown in Supplementary Figs. S1 to S8. (**a**) Viability of T cell cultures exposed to IK14800. (**b**) IL-2 levels in supernatant from T cell cultures exposed to IK14800. (**c**) IL-2 levels in supernatant from T cell cultures exposed to IK94000. (**d**) IL-2 levels in supernatant from T cell cultures exposed to IK00900. (**e**) IL-2 levels in supernatant from T cell cultures exposed to IK00904. (**f**) IL-2 levels in supernatant from PBMC cultures exposed to IK14800. (**g**) Percentage of CD4+ T cells expressing CD25 within PBMC cultures exposed to IK14800. (**h**) Percentage of CD8+ T cells expressing CD25 within PBMC cultures exposed to IK14800. (**i**) Percentage of CD4+ T cells expressing CD25 within PBMC cultures exposed to IK00900. (**j**) Percentage of CD8+ T cells expressing CD25 within PBMC cultures exposed to IK00900. (**k**) Expression of Ki67 in CD4+ T cells within PBMC cultures exposed to IK14800. (**l**) Percentage of CD4+ CD25+ Foxp3+ Treg cells within PBMC cultures exposed to IK14800. IK14004. (**m**) CD28-expressing CD4+ T cells within unstimulated, isolated CD4+ T cell cultures established from donors of all ages and exposed to IK14800 for 72 h. (**n**) CD28-expressing CD4+ T cells within anti-CD3/anti-CD28 stimulated, isolated CD4+ T cell cultures established from donors of all ages and exposed to IK14800. (**o**) Percentage of CD28-expressing CD4+ T cells within PBMC cultures established from elderly donors ≥ 70 years of age in the absence of peptide after 72 h. (**p**) Percentage of CD28-expressing CD4+ T cells within PBMC cultures established from elderly donors ≥ 70 years of age in the presence of peptide after 72 h. Data were analysed using repeated measures (RM) two-way ANOVA with Dunnett’s post-test comparing peptide with vehicle control and unpaired t test for young versus aged donors in the absence of peptide. *P < 0.05, **P < 0.01, ***P < 0.001, ****P < 0.0001.

Peripheral blood mononuclear cell (PBMC) and isolated CD3+ T cell cultures were stimulated with anti-CD3 and anti-CD3/anti-CD28 antibodies, respectively, and in preliminary flow cytometry studies we confirmed that IK14800 did not affect the viability of isolated CD3+ T cell cultures over 72 h (Fig. 1a). We next sought to examine the effect of IK14800 on IL-2 production by means of ELISA assays and IK14800 induced a dose-dependent increase in IL-2 production from isolated CD3+ T cell cultures after 72 h (Fig. 1b). Given that IK14800 comprises the sequence RSKAKNPLY (IK94000) linked at the C-terminus to nona-arginine (IK00900), these two peptide fragments were tested separately and neither IK94000 (Fig. 1c) nor IK00900 (Fig. 1d) enhanced IL-2 production. We have recently reported that IL-2 induction by the sequence RSKAKNPLYR is only seen when conjugated to a fatty acid cell-penetrating moiety^54^. To further exclude an effect of nona-arginine (R9) on IL-2 production we tested the effect of R9 (IK00900) linked at the C-terminus to four branched dodecanoic acid moieties and the lipidic peptide (IK00904) did not induce IL-2 production by isolated CD3+ T cells (Fig. 1e). We next sought to examine the effect of IK14800 in the presence of antigen-presenting cells (APCs) and IL-2 production was significantly enhanced in PBMC cultures after 24 h (Fig. 1f). Activated CD4+ T cells are the main producers of IL-2 leading to increased expression of the high-affinity IL-2 receptor subunit, IL-2Ra (CD25)^49^ and clonal expansion of T cells^55^. The addition of IK14800 to PBMC cultures led to increasing proportions of CD25-expressing CD4+ T cells (Fig. 1g) and CD8+ T cells (Fig. 1h) after 24 h and this effect was not observed in the presence of R9 (IK00900) for either CD4+ T cells (Fig. 1i) or CD8+ T cells (Fig. 1j). Moreover, addition of IK14800 to PBMC cultures enhanced the proliferative capacity of CD4+ T cells (Fig. 1k). Manipulating IL-2 shifts the balance between IL-2 effector cells and non-IL-2 producing immunosuppressive CD4+/CD25+/Foxp3+ regulatory T (Treg) cells^56^ and Tregs are sustained by low-dose IL-2 in contrast to high-dose IL-2 required for expansion of cytotoxic lymphocyte populations^57^. We therefore sought to assess the effect of IK14800 on the Treg cell population and the peptide induced a decrease in the percentage of Tregs within PBMC cultures after 48 h that was statistically significant at the highest concentration (Fig. 1l).

Optimal T cell activation requires signalling via the costimulatory receptor, CD28, that enhances T cell proliferation and production of multiple cytokines including IL-2 and IFN-γ^58^. To determine whether TCR stimulation was necessary for IK14800-mediated CD28 expression, we then compared the effect of IK14800 on isolated CD4+ T cell populations from donors across all ages in the presence/absence of TCR stimulation. Enhanced expression of CD28 on CD4+ T cells in the presence of peptide was not seen in the absence of TCR activation (Fig. 1m) but significantly increased after 72 h upon TCR activation with anti-CD3/anti-CD28 antibodies (Fig. 1n). Given that CD28 expression on CD4+ T cell declines with ageing^46,47^, we next sought to assess the effect of ageing on CD28 expression. In the absence of peptide, CD28 expression on CD4+ T cells within PBMC populations isolated from elderly donors (more than 70 years of age) was reduced by 50% compared with young donors (less than 30 years of age) (Fig. 1o). However, in the presence of IK14800, the proportion of CD28-expressing CD4+ T cells in aged donors increased at the two highest peptide concentrations after 72 h in culture (Fig. 1p).

### IK14800 enhances production of IFN-γ

**Figure 2:**
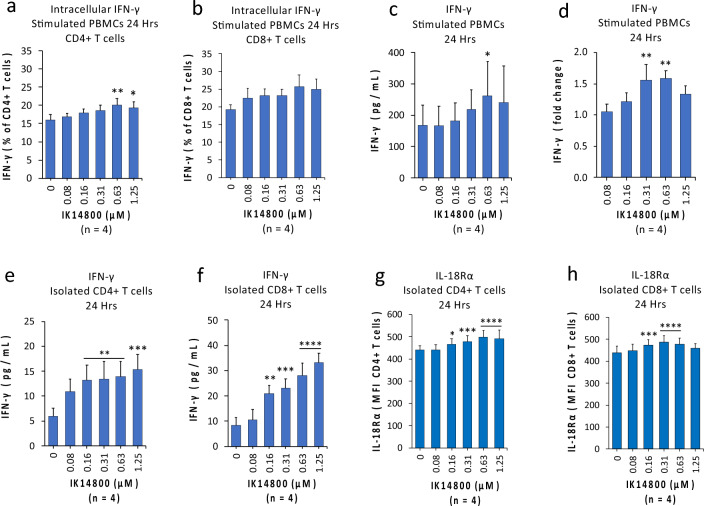
IK14800 enhances production of IFN-γ. PBMC were stimulated with either anti-CD3 antibody and isolated CD4+ T cells and CD8+ T cells isolated from CD3+ T cell populations were each stimulated with -CD3/anti-CD28 antibodies as described in the methods. Flow cytometry was used to determine intracellular IFN-γ expression in CD4+/CD8+ T cells within PBMC cultures and IFN-γ levels in supernatants from PBMC and isolated CD4+/CD8+ T cells cultures were assessed by ELISA. IL-18R expression on isolated CD4+/CD8+ T cells was determined by flow cytometry. Each tissue culture experiment was performed using triplicate wells (technical replicates) and repeated either three or four times (n = experimental replicates) as indicated below each panel. All error bars represent standard error of the mean (SEM). The culture duration was 24 h in all instances and the compound tested was IK14800. Flow cytometry data are shown as mean fluorescence intensity (MFI) and percentage values as indicated in the panels. Dot plots and gating strategies are shown in Supplementary Figs. S9 to S11. (**a**) Percentage of CD4+ T cells expressing intracellular IFN-γ within PBMC cultures. (**b**) Percentage of CD8+ T cells expressing intracellular IFN-γ within PBMC cultures. (**c**) IFN-γ levels in supernatant from PBMC cultures. (**d**) IFN-γ levels in supernatant from PBMC cultures expressed as fold-change between the lowest IK14800 concentration and higher concentrations with vehicle-treated cells normalised to 1. (**e**) IFN-γ levels in supernatant from isolated CD4+ T cell cultures. (**f**) IFN-γ levels in supernatant from isolated CD8+ T cell cultures. (**g**) Expression of IL-18Rα on isolated CD4+ T cell cultures. (**h**) Expression of IL-18Rα on isolated CD8+ T cell cultures. Data were analysed using repeated measures (RM) two-way ANOVA with Dunnett’s post-test comparing peptide with vehicle control. *P < 0.05, **P < 0.01, ***P < 0.001, ****P < 0.0001.

Tregs regulate CD4+ T helper cell type 1 (Th1) differentiation and suppress IFN-γ production during Th1 priming^59^. To determine whether IK14800-mediated Treg suppression (Fig. 1) was associated with changes in IFN-γ expression we used flow cytometry to assess the effect of IK14800 on intracellular IFN-γ expression in CD4+/CD8+ T cells within anti-CD3-stimulated PBMC cultures after 24 h. IK14800 induced a dose-dependent increase in the proportion of IFN-γ-expressing CD4+ T cells (Fig. 2a) and a slight, but not significant increase in IFN-γ-expressing CD8+ T cells (Fig. 2b). We next examined the effect of IK14800 on IFN-γ production from anti-CD3-stimulated PBMCs by means of ELISA and the peptide enhanced IFN-γ expression after 24 h shown as pg/mL (Fig. 2c) and fold increase (Fig. 2d). To further establish a role for IK14800 in IFN-γ production, we isolated CD4+ and CD8+ T cells and exposed the anti-CD3/anti-CD28-stimulated T cells to this peptide for 24 h. Increased IFN-γ levels within cell supernatants were observed for both isolated CD4+ T cell cultures (Fig. 2e) and isolated CD8+ T cell cultures (Fig. 2f) after 24 h. Given the role of IL-18 receptor (IL-18R) in mediating IFN-γ production^60^, we then examined the effect of IK14800 on IL-18R expression in anti-CD3/anti-CD28-stimulated CD4+ and CD8+ T cells and peptide-induced increases in IL-18R expression were observed in cultures of isolated CD4+ T cells (Fig. 2g) and CD8+ T cells (Fig. 2h) after 24 h.

### IK14800 elicits IL-12 production

**Figure 3:**
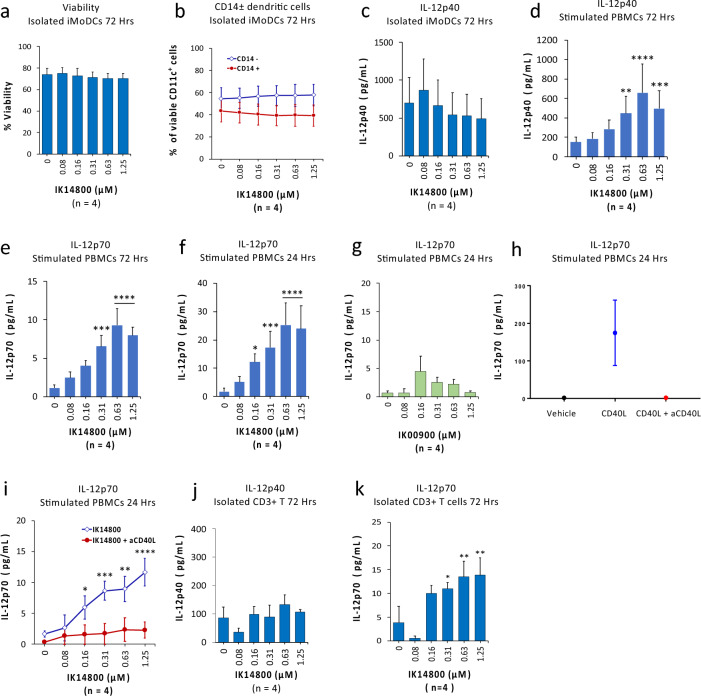
IK14800 elicits IL-12 production. Immature monocyte-derived dendritic cells (iMoDCs) were prepared as described in the methods. PBMC and iMoDCs were stimulated with anti-CD3 antibody and isolated CD3+ T cells stimulated with anti-CD3/anti-CD28 antibodies. Flow cytometry was used to determine the viability and phenotypic stability of iMoDCs. IL-12p40 and IL-12p70 levels in supernatants from iMoDC, PBMC and isolated CD3+ T cell cultures were assessed by ELISA. Each experiment was performed using triplicate wells (technical replicates) and repeated either three or four times (n = experimental replicates) as indicated below each panel. All error bars represent standard error of the mean (SEM). The culture duration was either for 24 or 72 h as indicated below each panel and the compounds tested were IK14800 and IK00900. Flow cytometry data are shown as percentage values as indicated in the panels. Dot plots and gating strategies are shown in Supplementary Fig. S12. (**a**) Viability of iMoDC cell cultures exposed to IK14800 for 72 h (**b**) Stability of iMoDC cell cultures exposed to IK14800 for 72 h. (**c**) IL-12p40 levels in supernatant from isolated iMoDC cultures exposed to IK14800 for 72 h. (**d**) IL-12p40 levels in supernatant from PBMC cultures exposed to IK14800 for 72 h. (**e**) IL-12p70 levels in supernatant from PBMC cultures exposed to IK14800 for 72 h. (**f**) IL-12p70 levels in supernatant from PBMC cultures exposed to IK14800 for 24 h. (**g**) IL-12p70 levels in supernatant from PBMC cultures exposed to IK00900 for 24 h. (**h**) IL-12p70 levels in supernatant from PBMC cultures exposed to recombinant CD40L (5 µg/mL) in the absence/presence of anti-CD40L-blocking antibody (5 µg/mL). (**i**) IL-12p70 levels in supernatant from PBMC cultures exposed to IK14800 for 24 h in the absence/presence of anti-CD40L-blocking antibody. (**j**) IL-12p40 levels in supernatant from isolated CD3+ T cell cultures exposed to IK14800 for 72 h. (**k**) IL-12p70 levels in supernatant from isolated CD3+ T cell cultures exposed to IK14800 for 72 h. Data were analysed using repeated measures (RM) two-way ANOVA with Dunnett’s post-test comparing peptide with vehicle control. *P < 0.05, **P < 0.01, ***P < 0.001, ****P < 0.0001 and for effect of rCD40L on IL-12p70 production by paired t-test.

Dendritic cells (DCs) are the main producers of IL-12 in response to microbial stimuli and interactions with T cells^44^ and we first examined the effect of IK14800 on viability and phenotype of isolated, anti-CD3-stimulated, immature monocyte-derived DCs (iMoDCs) by means of flow cytometry. IK14800 did not affect either viability of iMoDCs (Fig. 3a) or induce reversion of the cultured DCs to monocytes after 72 h (Fig. 3b). Maturation of DCs varies with their IL-12-producing capacity^61^ and is regulated by interactions between the CD40 ligand (CD40L) on T cells and CD40 expressed on DCs, respectively^45^. We therefore sought to compare the effect of IK14800 on IL-12p40 production by isolated iMoDCs versus stimulated PBMCs and no induction of IL-12p40 was observed in the absence of T cells after 72 h (Fig. 3c) in contrast to a dose-dependent increase in IL-12p40 production from stimulated PBMCs (Fig. 3d). Similarly, IK14800 enhanced the production of IL-12p70 by stimulated PBMCs after 72 h (Fig. 3e) which was more marked after the first 24 h (Fig. 3f) and, in contrast to IK14800, no IL-12p70 was induced by the nona-arginine sequence, R9 (IK00900) after 24 h (Fig. 3g). CD40L is expressed by activated T cells^45^ and to confirm a requirement for CD40L-CD40 interactions in IL-12 production from DCs we tested the effect of anti-CD40L neutralising antibody (5 µg/mL) on the ability of anti-CD3-stimulated PBMCs exposed to human recombinant CD40L (rCD40L; 5 µg/mL) to produce IL-12p70. Notably, rCD40L does not induce IL-12 production in unstimulated PBMC populations and in stimulated PBMCs the anti-CD40L blocking antibody completely inhibited rCD40L-induced IL-12p70 production (Fig. 3h). The CD40L blocking antibody also inhibited peptide-mediated production of IL-12p70 from anti-CD3-stimulated PBMC cultures although a small, non-significant increase in IL-12p70 was observed at higher peptide concentrations compared with negligible IL-12p70 levels from vehicle control-treated cells (Fig. 3i). T cells are also known to produce IL-12^28,54^ and we then examined the effect of IK14800 on production of the two IL-12 isoforms by isolated, anti-CD3/anti-CD28-stimulated T cells. Antigen-presenting cells produce IL-12p40 in vast excess compared with IL-12p70^62^ and, in contrast to no effect of peptide on IL-12p40 production by T cells (Fig. 3j), IK14800 induced a dose-dependent increase in the IL-12p70 level within culture supernatants from isolated T cell cultures after 72 h (Fig. 3k). This could not be accounted for by CD40L-CD40 interactions from contaminating DCs given the lack of effect on IL-12p40 production in isolated T cell cultures in contrast to peptide-mediated IL-12p40 production in PBMC cultures (Fig. 3d).

### IK14800 inhibits melanoma growth and re-invigorates exhausted CD4+ T cells

**Figure 4:**
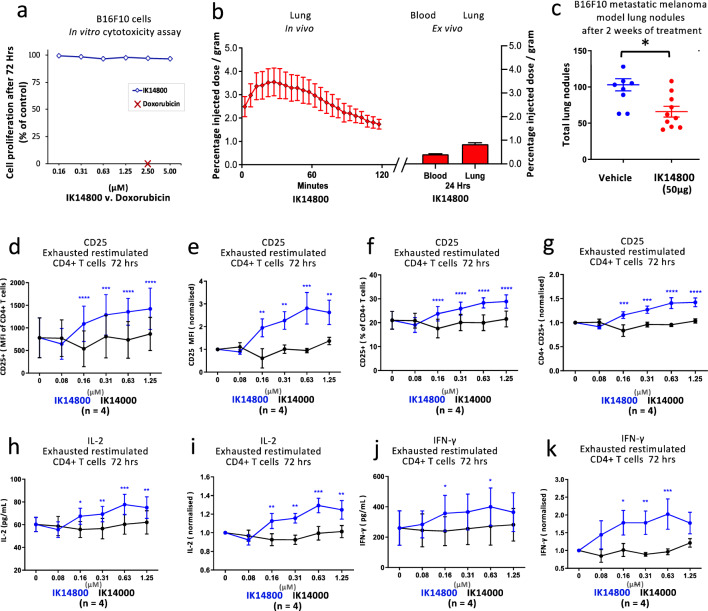
IK14800 inhibits melanoma growth and re-invigorates exhausted CD4+ T cells. Pharmacokinetic studies and experiments to determine the effect of IK14800 on the proliferation of B16F10 melanoma cells in vitro*,* growth of murine melanoma lung metastases and on CD25/IL-2/IFN-γ expression for murine-derived exhausted CD4+ T cells were undertaken as described in the methods. Three mice were used in pharmacokinetic studies. Data shown for the effect of IK14800 on B16F10 melanoma cells in vitro comprised 5 technical replicates per experiment from duplicate experiments. In murine exhausted CD4+ T cell studies, the effects of IK14800 on IL-2/IFN-γ production (ELISA) and CD25 expression (Flow cytometry) after 72 h were compared with a control peptide, IK14000, in 4 experimental replicates with triplicate technical replicates for each. All error bars represent standard error of the mean (SEM) and the dot plot/gating strategy for flow cytometry studies is shown in Supplementary Fig. S13. In the murine melanoma metastasis model 10 mice were assessed in the peptide (50 µg) and vehicle-treated cohorts. (**a**) Proliferation of B16F10 melanoma cells in vitro in the presence of IK14800 and Doxorubicin after 72 h. (**b**) Percentage uptake of ^64^Cu-NOTA-conjugated IK14800 in murine lung and blood over time up to 24 h. (**c**) B16F10 melanoma metastases within the lung after 2 weeks of treatment with IK14800. (**d**) CD25 expression in murine-derived exhausted CD4+ T cells comparing matched concentrations of IK14800 (blue) and the control peptide IK14000 (black) after 72 h. (**e**) CD25 expression in murine-derived exhausted CD4+ T cells comparing matched concentrations of IK14800 and the control peptide IK14000 with vehicle-treated cells normalised to 1. (**f**) Percentage of exhausted CD25-expressing CD4+ T cells comparing matched concentrations of IK14800 and IK14000 after 72 h. (**g**) Percentage of exhausted CD25-expressing CD4+ T cells comparing matched concentrations of IK14800 and IK14000 (normalised vehicle control values). (**h**) IL-2 levels in culture supernatants from exhausted CD4+ T cells comparing matched concentrations of IK14800 and IK14000 after 72 h. (**i**) IL-2 levels in culture supernatants from exhausted CD4+ T cells comparing matched concentrations of IK14800 and IK14000 (normalised vehicle control values). (**j**) IFN-γ levels in culture supernatants from exhausted CD4+ T cells comparing matched concentrations of IK14800 and IK14000 after 72 h. (**k**) IFN-γ levels in culture supernatants from exhausted CD4+ T cells comparing matched concentrations of IK14800 and IK14000 (normalised vehicle control values). Data from exhausted CD4+ T cell studies was analysed by means of two-way ANOVA with Sidak’s post-test comparing matched concentrations of IK14800 (blue) and the control peptide IK14000 (black). *P < 0.05, **P < 0.01, ***P < 0.001, ****P < 0.0001) and data from the murine melanoma study using a one-way ANOVA post-hoc comparison. *P < 0.05.

Metastatic melanoma is the most fatal type of skin cancer^63^ and the B16F10 murine melanoma cell line is a well-established resource to study primary and metastatic tumour growth^64^. To determine whether the immunomodulating effect of IK14800 may have relevance to this type of skin cancer, we compared its effect on growth of B16F10 melanoma cells in vitro and on growth of metastatic lung tumours*.* In preliminary experiments B16F10 melanoma cells in culture were exposed to IK14800 for 72 h and, in contrast to total cell kill in the presence of Doxorubicin (2.5 µM), this peptide did not induce any cytotoxicity at the highest concentration tested, i.e., 5 µM (Fig. 4a). We next performed pharmacokinetic studies using ^64^Cu-labelled NOTA-conjugated IK14800 and following intraperitoneal (IP) administration of peptide, retention of IK14800 in the lungs of C57BL/6 mice approximated 2% of the injected dose per gram of tissue two hours after injection (Fig. 4b). Ex vivo analysis of peptide retention in the lungs 24 h following injection showed 1% injected dose (ID)/g of lung tissue (Fig. 4b). Based on pharmacokinetic data and mid-nanomolar peptide concentrations required to induce a Th1-skewed cytokine response in vitro, we selected a dose of 50 µg of IK14800 (mass: 2,481 Daltons) to administer to tumour-bearing mice. Following IP administration of peptide three times per week for two weeks, the number of lung tumour nodules was significantly reduced in IK14800-treated mice (Fig. 4c).

Given that many of the known melanoma antigens are self-proteins expressed in normal melanocytes that can contribute to T cell exhaustion^65^**,** we then sought to determine whether IK14800 can re-activate exhausted CD4+ T cells after 72 h in culture. To address this possibility, we used a myelin basic protein (MBP)-Tracker Mouse model (see Methods) to compare the effect of IK14800 with a control peptide, RSKAKNPLYR (IK14000), that has been shown previously not to induce either IL-2/IFN-γ production or CD25 expression in human T cells isolated from healthy donors^54^. Using flow cytometry to compare matched peptide concentrations, a significantly greater induction of CD25 expression was observed in the presence of IK14800 compared with IK14000 (Fig. 4d) as also assessed when values for vehicle-treated cells were normalised to 1 (Fig. 4e). Similar findings were seen for the proportion of CD25-expressing CD4+ T cells (Fig. 4f) and when vehicle-treated cell values were normalised (Fig. 4g). IL-2 and IFN-γ levels in culture supernatants were assessed by means of ELISA. Comparing matched concentrations for the two peptides, IK14800 enhanced production of IL-2 compared with IK14000 (Fig. 4h) as also seen when vehicle-treated cell values were normalised (Fig. 4i). Similarly, enhanced production of IFN-γ was observed in the presence of IK14800 (Fig. 4j) and when assessed with normalised vehicle-treated cell values (Fig. 4k).

### IK14800 inhibits UVR-induced DNA damage

**Figure 5:**
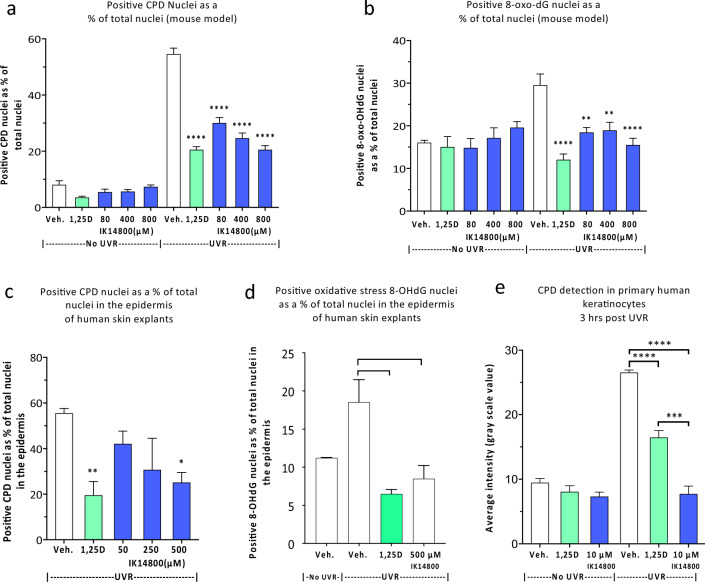
IK14800 inhibits UVR-induced DNA damage. Experiments to determine the effect of IK14800 on ultraviolet radiation-induced DNA damage in murine skin (in vivo), human skin explants (ex vivo) and primary human keratinocytes (in vitro) were undertaken as described in the methods. In all experiments peptide was applied immediately after exposure to UVR and DNA damage assessed 3 h later. Three mice per group were used for experiments involving UVR-administration. UVR-induced DNA damage in human tissue explants was assessed for two donors (3 technical replicates per donor) and for human keratinocytes cell cultures established from 3 skin biopsies. All error bars represent standard error of the mean (SEM). (**a**) Positive CPD nuclei in mouse skin from non-irradiated and irradiated mice exposed to topical application of either 1,25(OH)_2_D_3_ (10^–9^ M) or IK14800. (**b**) Positive 8-oxo-dG nuclei in mouse skin from non-irradiated and irradiated mice exposed to topical application of either 1,25(OH)_2_D_3_ (10^–9^ M) or IK14800. (**c**) Positive CPD nuclei in irradiated human skin explants in the presence of either 1,25(OH)_2_D_3_ (10^–9^ M) or IK14800 (representative data from one of two human skin explants). (**d**) Positive 8-oxo-dG nuclei in irradiated human skin explants in the presence of either 1,25(OH)_2_D_3_ (10^–9^ M) or IK14800 (representative data from one of the human skin explants). (**e**) Positive CPD nuclei in non-irradiated and irradiated primary human keratinocytes in the presence of either 1,25(OH)_2_D_3_ (10^–9^ M) or IK14800 3 h after irradiation. Data from UVR experiments were analysed using ANOVA with Tukey’s post-test. *P < 0.05, **P < 0.01, ***P < 0.001, ****P < 0.0001.

Given that prevention of UVR-induced immunosuppression by IL-12 depends on DNA repair^19^ and our findings of IK14800-induced IL-12p70 expression, we first sought to assess the effect of the peptide on UVR-induced DNA damage, i.e., formation of cyclobutane pyrimidine dimers (CPDs) in a murine model. The dorsal skin of Skh:hr1 hairless mice was exposed to solar-simulated combination of UVA and UVB radiation equal to three times the minimal erythemal dose^66^. Immediately following UVR, either 1,25(OH)_2_D_3_ (positive control; 10^−9^ M) or IK14800 at increasing concentrations (80–800 µM) was painted on to an area approximating 7 cm^2^ of dorsal skin of non-irradiated and irradiated mice and skin biopsies taken 3 h post-irradiation. Positive CPD nuclei were calculated as a percentage of total nuclei in the fixed, stained tissue sections and a dose-dependent decrease in CPD nuclei was observed for peptide treated mice which equalled the positive control at the highest concentration of IK14800 (Fig. 5a). Oxidative DNA damage generates DNA adducts such as 8-oxo-7,8-dihydro-2’-deoxyguanosine (8-OHdG) in the skin associated with UVR exposure^16^ and ageing^67^. To determine whether IK14800 inhibits DNA adduct formation following UVR exposure, the same concentrations of peptide were tested in the irradiated mouse model and the levels of 8-OHdG were lower when skin was treated with peptide (Fig. 5b). We next sought to determine whether IK14800 inhibits UVR-induced DNA damage in human skin explants. Skin obtained at elective surgery was cut into small pieces for placement into 96 well tissue culture plates in culture medium and treated immediately after irradiation with either 1,25(OH)_2_D_3_ (10^−9^ M) or IK14800 at increasing concentrations (50–500 µM). Significant inhibition of CPD positive nuclei was observed at the highest concentration of IK14800 (Fig. 5c) 3 h post-irradiation and levels of the DNA adduct (8-OHdG) were lower when human skin explants were treated with this peptide (Fig. 5d). To determine whether IK14800 can reduce UVR-induced DNA damage in keratinocytes, normal human epidermal keratinocytes established from skin biopsies were cultured in the presence of either peptide (10 µM) or 1,25(OH)_2_D_3_ (10^−9^ M) for 3 h following irradiation^68^. CPD formation was assessed by means of densitometry analysis on fixed, immune-stained cells and IK14800 inhibited CPD formation in keratinocytes to a significantly greater degree than 1,25(OH)_2_D_3_ (Fig. 5e).

### IK14800 inhibits UVR-induced MMP-1 expression and apoptosis in skin cells but not HaCaT cells

**Figure 6:**
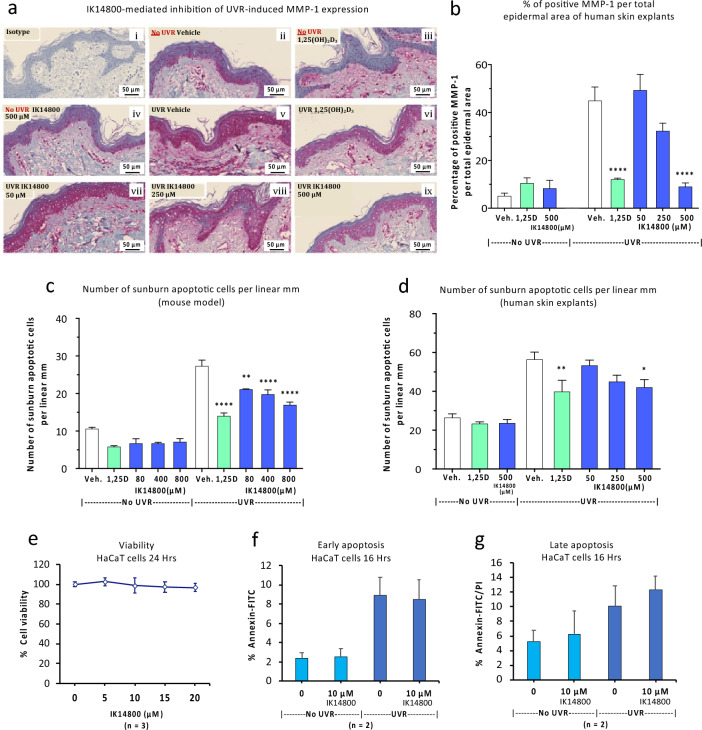
IK14800 inhibits UVR-induced MMP-1 expression and apoptosis in skin cells but not HaCaT cells. Experiments to determine the effect of IK14800 on UVR-induced MMP-1 expression were performed using human skin explants. Experiments to determine the effect of IK14800 on MMP-1 expression and UVR-induced apoptosis in murine skin and human skin explants are described in the methods. Human donor skin explants were exposed to peptide immediately after UVR In the collagenase-1 (MMP-1) study and MMP-1 expression assessed 24 h later. In the murine skin/human skin explant studies to assess apoptosis, peptide was applied immediately after irradiation and the apoptotic cell number assessed 3 h later. Three mice per group were used for experiments involving UVR-administration. UVR-induced apoptotic cells/MMP-1 expression in human tissue explants was assessed for two donors (3 technical replicates per donor). All error bars represent standard error of the mean (SEM). Assessment of UVB-induced apoptosis in HaCaT cells pre-treated with IK14800 was performed in duplicate and repeated twice with results expressed as mean ± SD. The Supplementary Annexin-FITC/PI flow cytometry data from HaCaT cells are shown in Fig. S14. (**a**: i-ix) Collagenase (MMP-1) expression in non-irradiated and irradiated human skin explants: (**a**-i) Collagenase (MMP-1) expression in non-irradiated human skin explants as assessed by staining with isotype control antibody; (**a**-ii) Collagenase expression in non-irradiated human skin explants exposed to vehicle control; (**a**-iii) Collagenase expression in non-irradiated human skin explants exposed to 1,25(OH)_2_D_3_; (**a**-iv) Collagenase expression in non-irradiated human skin explants exposed to IK14800 (500 µM); (**a**-v) Collagenase expression in irradiated human skin explants; (**a**-vi) Collagenase expression in irradiated human skin explants exposed to 1,25(OH)_2_D_3_; (**a**-vii) Collagenase expression in irradiated human skin explants exposed to IK14800 (50 µM); (**a**-viii) Collagenase expression in irradiated human skin explants exposed to IK14800 (250 µM); (**a**-ix) Collagenase expression in irradiated human skin explants exposed to IK14800 (500 µM). (**b**) Collagenase expression assessed by image analysis in non-irradiated and irradiated human skin explants exposed to 1,25(OH)_2_D_3_ and IK14800 for immunostaining data shown in (**a**). (**c**) Positively stained apoptotic cells in skin samples from non-irradiated and irradiated mice exposed to topical application of either 1,25(OH)_2_D_3_ (10^–9^ M) or IK14800. (**d**) Positively stained apoptotic cells in non-irradiated and irradiated human skin explants in the presence of either 1,25(OH)_2_D_3_ (10^–9^ M) or IK14800 (representative data from one of two human skin explants). (**e**) Viability of HaCaT cells exposed to IK14800 for 24 h. (**f**) UVB-induced early apoptosis in HaCaT cells exposed to IK14800 (10 µM) for 24 h pre-irradiation and assessed 16 h after exposure to UVB. (**g**) UVB-induced late apoptosis in HaCaT cells exposed to IK14800 (10 µM) for 24 h pre-irradiation and assessed 16 h after exposure to UVB. Data from UVR experiments using murine and human tissues/cells were analysed by means of ANOVA with Tukey’s post-test. *P < 0.05, **P < 0.01, ***P < 0.001, ****P < 0.0001.

Both UVA and UVB induce expression of matrix metalloproteinase-1 (interstitial collagenase) associated with photo-carcinogenesis which serves to promote tissue invasion by melanoma and non-melanoma skin cancers (basal cell and squamous cell cancers)^69,70^. Accordingly, we sought to determine the effect of IK14800 on UVR-induced MMP-1 expression in human skin explants. Histochemical staining of normal skin with an isotype-matched control antibody showed no staining for MMP-1 (Fig. 6a–i). In the absence of UVR, patchy MMP-1 expression was seen in the deeper epidermal layer of skin explants in the absence of compounds (Fig. 6a-ii) and in the presence of either 1,25(OH)_2_D_3_ (10^–9^ M) (Fig. 6a-iii) or IK14800 at the highest concentration (500 µM) (Fig. 6a-iv). Exposure of skin explants to UVR markedly induced MMP-1 expression (Fig. 6a–v) which was inhibited in the presence of 1,25(OH)_2_D_3_ (Fig. 6a-vi) while no inhibition of MMP-1 expression was observed in the presence of IK14800 at the lowest concentration (50 µM) (Fig. 6a-vii). However, slightly less intense staining for MMP-1 was observed in the presence of 250 µM IK14800 (Fig. 6a-viii). At the highest concentration of peptide (500 µM), inhibition of MMP-1 expression (Fig. 6a-ix) was equivalent to that seen in the presence of 1,25(OH)_2_D_3_ (Fig. 6a-vi) and image analysis by means of the Metamorph program^70^ confirmed the visible effects (Fig. 6b).

Sunburn cells are keratinocytes undergoing apoptosis after they have received a physiological UVB dose that damages their DNA^71^. Using the identical protocol to that used for CPD studies following irradiation and compound application to Skh:hr1 mice and human skin explants we compared the number of apoptotic sunburn cells per linear mm in the epidermis of both models in the absence and presence of UVR. All concentrations of peptide significantly inhibited UVR-induced apoptosis in a dose-dependent manner when applied topically to dorsal skin of Skh:hr1 mice immediately after UVR (Fig. 6c) and, in human skin explants, the level of inhibition of sunburn cells at the highest peptide concentration approximated that seen for 1,25(OH)_2_D_3_ (Fig. 6d). The link between IL-12 and inhibition of apoptosis in keratinocytes exposed to UVB has been reported previously for the spontaneously transformed human keratinocyte cell line, HaCaT^18^ and HaCaT cells serve as a model system for Vitamin D3 metabolism in human skin^72^. In a preliminary study we confirmed that IK14800 does not affect viability of cultured HaCaT cells (Fig. 6e). Given that pre-treatment of keratinocytes with IL-12 prior to UVR appears necessary to elicit full IL-12 effects^18,21^, we treated HaCaT cells with IK14800 at a concentration (10 µM) that had been found to effectively inhibit CPD formation in UVR-exposed human keratinocytes (Fig. 5e). Surprisingly, exposure of HaCaT cells to peptide for 24 h prior to treatment with UVB did not inhibit either early apoptosis (Fig. 6f) or late apoptosis (Fig. 6g) as assessed 16 h after exposure to UVB.

## Discussion

Herein we present evidence of immunomodulation by a novel non-naturally occurring peptide (IK14800) comprising RSKAKNPLYR linked to a cell-penetrating octa-arginine sequence^53^. Hence, IK14800, effectively comprises a nona-arginine (R9) moiety linked to the amino acid sequence RSKAKNPLY. Nona-arginine has been shown to be an efficacious cell-penetrating peptide for intracellular delivery of cargoes^73^ and the effects of IK14800 on cytokine expression cannot be ascribed to biological activity of the R9 moiety. However, the benefit of conjugating a polyarginine sequence to an impermeable peptide cargo to achieve rapid cell entry, e.g., within one hour, has been highlighted^74^. Nevertheless, we have not established where within T cells IK14800 exerts its effects. For example, we do not know whether IK14800 enters only the cytosol or also the nucleus given that octa-arginine alone or with a cargo has been shown to efficiently localise to the nucleus in macrophages^75^. The RSKAK motif within IK14800 may play a role in gene induction given that a homologous stretch of amino acids within the nuclear localisation sequence of tumour inhibitor of growth 4 (ING4) protein, i.e., RARSK, is known to bind to p53 located in the nucleus^76^.

Nucleotide excision repair (NER) following UV light-induced DNA damage is a highly complex process involving multiple factors^77^ that are linked to IL-12^17–20^. Notably, the Vitamin D receptor is also involved in normal DNA repair although the exact mechanisms by which it acts remain unclear^78^. The effects of 1,25 dihydroxyvitamin D_3_ (1,25 (OH)_2_D_3_) on NER are thought to involve, at least in part, reduced nitrosylation of DNA repair enzymes^66^, as well as increased energy availability^68^ and increased access of repair proteins^78^. However, in the case of Vitamin D3, it seems unlikely that IL-12 is involved because Vitamin D3 inhibits IL-12 production by antigen-presenting cells^79^. Nevertheless, we observed similar effects between the positive control, 1,25 (OH)_2_D_3_, and IK14800 in terms of reduction in UVR-induced CPDs, 8-OHdGs and sunburn (apoptotic) cells when applied topically on murine skin and to human skin explants in vitro suggesting that IK14800 may exert an effect on the DNA damage repair pathway. Interestingly, IL-12-mediated protection from UVB-induced apoptosis has been reported in HaCaT cells^18^ that are known to produce IL-12p40/p70^80,81^. However, an unexpected finding from our study was that exposure of HaCaT cells to IK14800 did not reduce UVB-induced apoptosis. This raises the possibility that IK14800 affects NER in human skin cells in an IL-12-independent manner. For example, toll-like receptor (TLR) signalling may lead to increased or decreased DNA repair depending on context^82^ and TLR-4 deficiency enhances repair of UVR-induced cutaneous DNA damage via NER^83^. Notably, HaCaT cells express TLR4 in contrast to its absence in primary human keratinocytes^84^, and whether peptide-mediated DNA repair in human skin cells is determined by their TLR expression profile remains to be established. In addition, 1,25 (OH)_2_D_3_ regulates non-classical pathways implicated in UVR-induced DNA damage such as inhibition of phosphorylation of cyclic AMP response binding element protein (CREB)^16^ and an effect of IK14800 on CREB phosphorylation also warrants further investigation.

The cytokine IL-12 is one of the major players in orchestrating both innate and immune responses and polarises T cells into a type 1 helper T (Th1) effector cell phenotype^39^ that can counteract UVB-induced immunosuppression^33,34^. Much higher levels of IL-12p40 than IL-12p70 are normally expressed^62^ and IL-12p40 competitively inhibits the effects of the IL-12p70 heterodimer^85^. Notably, synthesis of the p35 subunit of the IL-12p70 isoform has been proposed as the rate-limiting step for IL-12 production because of low abundance of the transcript under steady-state conditions^86^. UVB irradiation is known to impair Th1-mediated immune responses in vivo by specific suppression of systemic IL-12p70 production^87^. In the absence of exposure to UVR, we show that IK14800 induces secretion of the biologically active IL-12p70 isoform as opposed to IL-12p40 in isolated T cell cultures (Fig. 3) indicating that T cell-mediated licensing of DCs is not the only pathway involved in IL-12p70 production. Notably, epidermal LCs become depleted upon exposure of skin to UVR^30^ and murine LCs exposed to UVB are converted from immunogenic to tolerogenic APCs^88^. Hence, an alternative source of IL-12p70 such as from T cells may be beneficial in countering UVR-induced immunosuppression. Interestingly, IL-12p70 levels in serum are significantly reduced in UVB-irradiated mice whereas no effect is seen in IL-12p40 levels^87^. Hence, it is this reduction of the bioactive heterodimeric form of IL-12, i.e., IL-12p70, that likely explains the reduction in systemic Th1 response after UVB irradiation^87^.

CD4+ T cell-derived secretion of the characteristic tumoricidal cytokine, IFN-γ, has been acknowledged as a read-out of increased anti-tumour CD4+ responses^89^ and IL-12 also upregulates expression of the IL-18 receptor (IL-18Rα) which leads to enhanced IFN-γ production^90^. IK14800 enhances both IL-18Rα expression and IFN-γ production by isolated CD4+ and CD8+ T cells and, interestingly, IFN-γ liniment protects mice from UVB-induced skin damage consistent with the deficiency in Th1-associated cytokines such as IL-2, IL-12 and IFN-γ upon UVB exposure^23^. Moreover, syngeneic tumour-bearing mice exposed to chronic UVR demonstrate an inability of host tumour-draining lymph node cells to mount an IFN-γ response to tumour antigens^91^. While we do not know whether the relatively small increase in expression of IL-18Rα in isolated T cells exposed to IK14800 has biological significance, it nevertheless raises the possibility of either additive or synergistic effects with IL-12 given that IL-18Rα expression is known to contribute to the DNA repair process^92^. However, CD4+ T cell-mediated IFN-γ production can be suppressed by CD4+ CD25+ Foxp3-expressing T regulatory cells (Tregs)^59^ and, surprisingly, IK14800-mediated suppression of Tregs was not more marked. The IL-12p70 heterodimer promotes induction of Tregs^93^ in contrast to their suppression by IL-12p40^94^. Notwithstanding much larger increases in IL-12p40 compared with IL-12p70 secretion from PBMCs in the presence of peptide (Fig. 3), we suggest that peptide-mediated induction of IL-12p70, but not IL-12p40 by isolated T cells, acts to limit potential anti-Treg effects induced by IL-12p40 in the presence of IK14800.

T cell activation requires specific antigen recognition by the TCR plus a second signal from co-stimulatory molecules and lack of expression of the classic co-stimulatory molecule, CD28^95^, is common with increasing age in healthy individuals^52^. Functional CD28 expression in a proportion of CD4+ CD28^null^ T cell clones can be induced by recombinant IL-12 combined with anti-CD3 stimulation which peaks after 6 days in culture^52^. In the absence of peptide, we show a marked reduction of CD28-expressing CD4+ T cells isolated from elderly donors compared with young donors as expected and peptide-induced CD28 expression in CD4+ T cells from donors across all ages is dependent on the ability to activate the TCR. In elderly individuals aged more than 70 years, higher concentrations of peptide enhanced the proportion of CD28-expressing CD4+ T cells after 72 h in culture and whether this is related to peptide-induced IL-12p70 production in the elderly has not been established. However, it has been proposed that in the context of an ageing immune system, re-expression of CD28 may be desirable to restore immunocompetence^52^ which is relevant to the age-linked incidence of skin cancers^96–98^.

The CD40 ligand, CD40L, expressed on T cells interacts with the CD40 co-stimulatory receptor on DCs^45^ and is upregulated by CD28 signalling^52^. CD40L is considered critical for IL-12 production by antigen-presenting cells^43^ and, thereby, IFN-γ production by T cells in an IL-12-dependent manner^99^. However, a component of IFN-γ production has also been shown to be dependent on IL-2-signalling in addition to dependency on IL-12^99^. We observed that constitutive expression of CD40L in T cells is clearly necessary for IL-12p70 production by APCs as shown by inhibition of IL-12p70 production in the presence of anti-CD40L antibody irrespective of the absence or presence of peptide. However, IK14800 also enhances production of IL-12p70 in isolated T cells in an environment lacking CD40-expressing DCs, i.e., in the absence of CD40L-CD40 interactions. Contaminating DCs were unlikely to account for this observation of increased IL-12p70 production because IK14800 did not enhance IL-12p40 secretion which is normally produced in such vast excess^62^ as shown by the relatively high basal level of IL-12p40 in PBMC cultures compared with IL-12p70 (Fig. 3). Taken together, we suggest that in the absence of T cell–DC contact, IK14800-mediated IFN-γ production involves both IL-2 and IL-12p70 signalling pathways.

We acknowledge several limitations to our study. For example, we have not assessed the effect of IK14800 on IL-12 expression in either non-UV-irradiated or irradiated murine and human skin models. Neither have we assessed the effect of IK14800 on development of skin cancer and future studies will seek to determine whether topically applied peptide can suppress UVR-induced keratinocyte-derived skin cancers in Skh:hr1 hairless mice. Notably, UVR induction of cutaneous melanoma involves a variety of complex mechanisms that include species differences between humans and mice with respect to melanocyte distribution and this requires careful choice of the appropriate murine model^100^. Furthermore, we have not performed NER gene profiling in keratinocytes and immune cells derived from wild-type and *XPA* knockout cells exposed to peptide. Since disruption of *XPA* results in mice that are severely deficient in NER^101^, testing the effect of peptide on DNA damage in *XPA* knockout mice may also be informative.

In the poorly immunogenic, highly aggressive B16F10 melanoma model^102^ used in the present study inhibition of melanoma metastases by IK14800 appears to be due to peptide-mediated immunomodulation. For example, IK14800-mediated production of IL-2, IL-12 and IFN-γ by human immune cells contrasts markedly with the immunosuppressive environment associated with melanoma^24,25^. Hence, IL-2 administration^103^ and IL-12 gene therapy^104^ have shown beneficial effects against B16F10 lung metastases as has IFN-γ in preventing melanoma growth^105^. Furthermore, T cells exposed to self/melanoma antigens in healthy tissues develop an exhaustion-like phenotype^65^. IK14800 re-invigorates murine-derived exhausted CD4+ T cells obtained from a non-tumour murine model and when taken together with lack of in vitro cytotoxicity displayed by IK14800 at a ten-fold higher peptide concentration in B16F10 melanoma cell cultures than achievable in vivo, this lends support to the notion that IK14800 suppresses melanoma progression via immunomodulation.

Approximately twenty percent of all skin cancers comprise aggressively invasive squamous cell cancers^106^ and the mortality rate from cutaneous melanoma is linked to more deeply invasive tumours^107,108^. The peptide clearly did not act as a sunscreen in the current study using mice, human skin explants or primary human keratinocytes, as it was applied immediately after UV exposure. UV-induced DNA damage initiates release of MMP-1 in human skin^70^ and antisense RNA for MMP-I has been shown to suppress basement membrane type IV collagen degradation and cell invasion by human melanoma cells^109^. Both UVA and UVB induce MMP-1 (interstitial collagenase) in skin associated with photo-carcinogenesis and MMP-1 is secreted mainly by skin keratinocytes and dermal fibroblasts which then promotes invasion by breaking down interstitial collagen barriers^69,110^. This process is preventable when keratinocytes are treated with DNA repair endonucleases^70^ and the inhibitory effect of IK14800 on UVR-induced MMP-1 expression in human skin explants highlights a potential use for this peptide in not only limiting tissue invasion associated with photo-carcinogenesis but also in slowing the effects of photoageing.

Whether IK14800 can block the effects of UVR spectra by acting as an ultraviolet light filter^111^ is not known. Sunscreens are designed to primarily prevent UVB-associated skin burning and damage^112^ and may contribute to preventing UVR-induced melanoma^100,112^. Enhancing NER by either cytokines^20^ or topical application of bacterial DNA repair enzymes^17^ was proposed some years ago. However, to date there has been a lack of randomised controlled trials demonstrating the superiority of sunscreens with DNA repair enzymes over conventional sunscreens and photoprotection by the application of conventional sunscreen products is of no value once DNA damage has occurred^113^. Given the alarming increase of UVR due to depletion of the stratospheric ozone layer^113^ supplements to existing strategies aimed at preventing skin cancer continue to remain an unmet need^20^.

In summary, we have developed an immunomodulating peptide that promotes an immune response relevant to overcoming UVR-induced immunosuppression and which also reduces UVR-induced DNA damage by mechanisms yet to be determined. Peptide-enhanced IFN-γ and IL-12p70 production by T cells in the absence of DCs suggests that the effect of IK14800 may not necessarily require cross-priming of DCs. This has relevance to depletion of Langerhans cells within skin upon exposure to UVR. Moreover, re-invigoration of exhausted T cells and inhibition of UVR-induced MMP-1 production highlight mechanisms whereby the peptide may subvert progression of skin cancers, particularly melanoma. Taken together, IK14800 offers an opportunity to gain further insight into how UVR and ageing contribute to the rising incidence of skin cancers.

## Materials and methods

### Peptide synthesis

All peptides were manufactured by Auspep (Melbourne, Australia). The unmodified sequence IK14800 (RSKAKNPLYRRRRRRRRR-NH_2_; mass 2.481 kDa) and its component parts, i.e., IK94000 (RSKAKNPLY-NH_2_), RSKAKNPLYR-NH_2_ (IK14000) and IK00900 (RRRRRRRRR-NH_2_) were assembled by solid phase, peptide synthesis using Fmoc protected amino acid building blocks on Rink AM resin. The modified sequence, NOTA-IK14800 (NOTA-RSKAKNPLYRRRRRRRRR-NH_2_), was prepared by activating 1,4,7-Triazacyclononane-1,4-bis-tert-butylacetate-7 acetic acid (Macrocyclics. Inc, Plaino TX, USA) and coupling this to the N-terminal of the IK14800 sequence. The lipidic peptide, IK00904 (RRRRRRRRR-[(2)Adod]_4_ -NH_2_), was synthesised by the sequential addition of four residues of (2)Adod [(*S)*-2-aminododecanoic acid] using Fmoc-(2)Adod-OH (Watanabe Chemical Industries LTD, Japan) to the Rink AM resin followed by the nine arginine residues. Once assembled, all peptides were globally deprotected and cleaved from the resin liberating the crude, C-terminally amidated peptides. These were purified, and salt exchanged to an acetate counter-ion by RP-HPLC (C18) to a purity of > 95%. The product structures were confirmed by mass spectroscopy and amino acid analyses.

### B16F10 in vitro study

B16F10 melanoma cells (sourced from the American Type Culture Collection) were seeded into 96-well plates (1000 cells/well) in complete cell culture medium and allowed to attach for 24 h (37 °C, 5% CO_2_ in air). Next, an equal volume of either cell culture medium only, or 2 × concentration of drug dissolved in cell culture medium, was added to each of 5 replicate wells (technical replicates) to expose cells to concentrations of IK14800 in the dose range from 0 to 5 µM. Cells were cultured for 72 h in the presence of either IK14800 or Doxorubicin (2.5 µM; positive control) and then the cell culture medium was removed and the attached cells fixed in ice-cold trichloroacetic acid. Fixed cells were stained with Sulforhodamine B (SRB) and then washed with 1% acetic acid to remove unbound dye. The retained dye was solubilised in 10 mM Tris base solution and the absorbance at 550 nm was measured with the baseline (media only without cells) subtracted. The data were normalised between the maximum proliferation (100%, cells with no drug) and the starting cell density (0%, cells before addition of drug). Each experiment was performed on two independent occasions (biological replicates).

### Animal ethics

B16F10 tumour growth, pharmacokinetic analyses and ultraviolet radiation murine studies.

*Accordance*: All methods were carried out in accordance with relevant guidelines and regulations.

*Arrive guidelines*: All methods are reported in accordance with ARRIVE guidelines (https://arriveguidelines.org).

### B16F10 in vivo study

Mouse experiments were approved by the Peter MacCallum Cancer Centre Animal Experimentation Ethics Committee (Ethics approval number E592). Twenty female C57Bl/6 mice (WEHI, Age 9 weeks) were inoculated intravenously with 2 × 10^5^ B16F10 cells in phosphate buffered saline (PBS) on Day 1. Mice were then randomised into two groups of 10 mice: MilliQ water (vehicle); and 50 μg IK14800 in 100 μL MilliQ water. Dosing was performed via intraperitoneal (IP) injection three-times-weekly for two weeks (on Days 1, 3, 5, 8, 10 and 12). Mice were monitored for general health and body weight on each dosing day. On Day 15 mice were euthanised by CO_2_ asphyxiation, the lungs were removed and rinsed in PBS before being fixed in Fekete’s solution and counting all lung tumour nodules (black and white).

### Pharmacokinetic analyses

Mouse studies were approved by the University of Queensland Animal Ethics Committee (AEC Approval no. AIBN/CAI/530/15/ARC/NHMRC). IK14800 was dissolved in MilliQ water at 1 mg/mL and labelled with ^64^Cu at 1000-fold excess of peptide in acetate buffer (~ 50 mM, pH 5.5, 37 C, 1 h with shaking). This resulted in radio-pure products with no free copper detected by radio-TLC. Radio-labelled peptides were diluted with MilliQ water and 50 µL of H_2_O (containing acetate buffer) were injected into the intraperitoneal cavity (IP) of C57BL/6 mice (n = 3). Mice were anaesthetised using isofluorane in O_2_ and imaged using PET-CT for 2 h following injection and then again at approximately 24 h after injection. Blood samples were taken by tail snip following the initial imaging and activity measured by gamma counter. After imaging at 24 h post injection, a final blood sample was collected. Mice were euthanized by cervical dislocation and lung/blood samples collected. Activity of each sample was then measured via a gamma counter and the activity present was normalized to tissue weight to provide percent injected dose per gram (% ID/g).

### Cell cultures for flow cytometry and ELISA assays

All methods were carried out in accordance with relevant guidelines and regulations. Buffy coat samples from healthy human donors were obtained from Research Donors Limited via Cambridge BioScience. Ethics approval was granted by the Black Country Research Ethics Committee under REC reference 19/WM/0260. Informed consent for buffy coat samples was obtained from all subjects and/or their legal guardians in accordance with the Helsinki Declaration.

Preparations of peripheral blood mononuclear cells (PBMCs) and isolated T cells from buffy coat samples were performed using SepMate tubes, EasySep selection and enrichment kits, Lymphoprep, RoboSep Buffer, and EasySep magnets (STEMCELL Company). PBMCs were resuspended in RPMI-10 (RPMI-1640; ThermoFisher) supplemented with 10% heat inactivated Foetal Bovine Serum (LabTech), 100 U/mL penicillin, 100 µg/mL streptomycin (ThermoFisher), 2 mM L-glutamine (ThermoFisher), and 50 µM β-mercaptoethanol (ThermoFisher) at 1 × 10^6^ cells/mL and plated at a density of 1 × 10^5^ per well (100 µL) in 96-well, flat-bottom culture plates. PBMCs were stimulated with 1 µg/mL of soluble anti-CD3 (BioLegend). Peptides IK14800, IK14000, IK94000, IK00900 and the lipidic peptide, IK00904, were solubilised as a 1 mM stock solution in sterile milliQ water (Lonza) and added to wells at a final volume of 50 µL per well together with soluble anti-CD3 (1 µg/mL final, 50 µL per well) (BioLegend Lot no. B235453). To test the effect of peptides, cells were cultured for 24–72 h at 37 °C and 5% CO_2_ and vehicle controls in peptide-based experiments comprised 0.13% sterile milliQ water in culture medium.

CD3+ T cells were isolated from PBMCs by negative selection using immune-magnetic separation (Stem cell kits), resuspended in complete medium as used for PBMCs at 0.5 × 10^6^/mL and plated at a density of 5 × 10^4^ per well (100 µL) in 96-well, flat bottom culture plates. CD4^+^ and CD8^+^ T cell populations were isolated by immunomagnetic separation and resuspended in RPMI-10 at 0.5 × 10^6^/mL with a plating density of 0.5 × 10^5^ per well. The peptides IK14800, IK94000, IK14000, IK00900 and IK00904 were added to wells at a final volume of 50 µL per well, together with anti-CD3 anti-CD28 coated Dynabeads (ThermoFisher) at a 4:1 cell:bead ratio (1.25 × 10^4^/well, 50 µL volume) and cells cultured for 72 h at 37 °C and 5% CO_2_. Dendritic cells (DCs) were induced from CD14+ monocytes (without CD16 depletion) that had been isolated from PBMCs using immune-magnetic separation (positive selection) (Stem cell kit) and cultured with Mo-DC differentiation medium (Miltenyi Biotec) for seven days. At the end of the 7-day period the induced CD14^neg^CD11c+ DCs were classed as immature monocyte-derived dendritic cells (iMoDCs) and cultured in the presence of anti-CD3 antibody.

### Flow cytometry

Staining was performed to determine cell viability (Flexible Viability Dye eFluor™ 780; ThermoFisher) and expression of extracellular/intracellular markers using the following fluorescently-labelled antibodies against human proteins: CD4 (FITC Mab OKT4; ThermoFisher), CD8 (BV711/clone SK1, BioLegend), CD25 (PE/Cy7; BioLegend), CD28 (PE/Cy7; BioLegend); CD127 (eFluor450/eBioRDR5; ThermoFisher) within different cell populations. For intracellular staining Brefeldin (3 µg/mL)(Life Technologies) was added to cultures 4 h prior to flow cytometry and intracellular staining for Ki67 (Alexa Fluor 488; BioLegend) within CD4+ /CD8+ T cells and iMoDC populations as well as staining for intracellular IFN-γ (PE clone B27; BioLegend). Staining for T regulatory (Treg) cells was performed using anti-Foxp3 (PE conjugate; BioLegend) within CD4+/CD127^low^/CD25+ T cells following fixation and permeabilization (Foxp3 transcription factor fixation buffer; ThermoFisher).

### ELISA assays

Supernatants were obtained from PBMC and isolated CD3+ T cells cultures to assess production of IL-2, IL-12p70, IL-10 and IFN-γ (ThermoFisher kits) and IL-12p40 (BioLegend). Recombinant CD40L was purchased from BioLegend (Lot no. B247427) as was the anti-CD40L blocking antibody (Lot #B213441). ELISA plates were read at 450 nm using an Infinite F50 (Tecan) absorbance reader and Magellan™ reader control and data analysis software. Flow cytometry data was exported as FCS files from Attune™ NxT software and analysed using FlowJo™ software, from which data were tabulated for export to Microsoft Excel.

### Exhausted CD4+ T cell assay

Aquila/Concept Life Sciences had established a proprietary method for assessing murine CD4+ T cell exhaustion in vitro using Tg4-Ly5.1 MBP-Tracker Mice obtained from the University of Edinburgh. Spleens were removed from transgenic B10PLxC57BL/6 mice and processed to generate a single cell suspension of splenocytes. Myelin basic protein (MBP)-Tracker splenocytes were resuspended at 3 × 10^6^/mL and stimulated for 72 h at 37 °C, 5% CO_2_, with altered peptide ligand (APL)-MBP (to generate exhausted cells). Following stimulation, T cells were purified by Ficoll density gradient, and subsequently re-plated at 2 × 10^6^/mL in 20U/mL IL-2 for four days. At the end of this rest period, cells were resuspended (4 × 10^5^/mL, final 2 × 10^4^ per well) and restimulated using irradiated APCs (from B10PLxC57BL/6 mice, 4 × 10^6^/mL, final concentration of 2 × 10^5^ cells per well) plus a single dose of APL-MBP peptide together with IK14800 peptide for 72 h. Supernatants were sampled from culture wells for assessment of IL-2 and IFN-γ by ELISA (eBioScience, Lot. # 4,280,695 and Lot #. 4,308,729, respectively) and flow cytometry used for assessment of CD25 (PE/Cy7; BioLegend).

### Ultraviolet radiation murine studies

The irradiation experiments were approved by the Animal Ethics Committee of the University of Sydney (K22/3-2005/4/4089). Female Skh:hr1 hairless mice were maintained in wire-topped plastic boxes at 23–25 °C on compressed paper bedding from Fibrecycle Pty. Ltd. (Mudgeeraba, Australia). Mice were fed Gordon Rat and Mouse Pellets (Yandeera, Australia) and tap water ad libitum. The solar simulated UVR source was one fluorescent UVB tube (Philips TL40W 12R/S, Eindhoven, The Netherlands) flanked by 6 UVA tubes (Hitachi 40W F40T 10/BL, Tokyo, Japan) and was filtered through 0.125 mm cellulose acetate sheeting (Grafix Plastics, Cleveland, OH). Animals were randomly allocated to treatment groups of 3 for studies involving histological analyses. UV-irradiated mice were subjected to a single exposure equal to three times the minimal erythemal dose (MED) of UVR (3.98 kJ/m^2^ UVB and 63.8 kJ/m^2^ UVA) for histological studies. Immediately after irradiation mice were treated topically on the dorsal surface with either vehicle, 1,25(OH)_2_D_3_, or IK14800. Stock solutions of 1,25(OH)_2_D_3_ were dissolved in ethanol and those of IK14800 were dissolved in water and diluted in a base lotion containing ethanol, propylene glycol and water to a final solvent ratio of 2:1:1 respectively. Treatments were applied topically (100 µL) on the highest part of the back and were either vehicle (base lotion) only, or vehicle containing 1,25(OH)_2_D_3_ (11.4 pmol/cm^2^ as a positive control) or IK14800 (dose in the range between 20 and 200 µg in 100 µL).

Biopsies were taken from UV-irradiated dorsal skin 3 h post-UVR and fixed in Histochoice fixative (Amresco, Solon OH) for 6 h. Skin samples were paraffin-embedded and 5 µm sections were cut for all analyses. DNA damage in the form of cyclobutane pyrimidine dimers (CPDs) and 8-oxo-2'-deoxyguanosine (8oxodG; 8-OHdG) was detected by immunohistochemistry and image analysis as previously described^16^. Slides were deparaffinised and rehydrated in a series of graded ethanol solutions. Antigen retrieval was performed using Proteinase K at 37 °C for 30 min, followed by treatment of sections with 2N HCl (in 70% ethanol) for 15 min, and then followed by 50 mM Tris buffer for 15 min. For 8-OHdG assessment the slides were further treated with RNase A at 200 µg/mL (Amresco, Ohio, USA) at 37 °C for 30 min. Subsequent steps were carried out using the Dako Animal Research Kit using the method prescribed by the manufacturer (Dako, Glostrup, Denmark). Thymine dimers are the major type of CPD and the anti-thymine dimer antibody (Sigma-Aldrich, Missouri, USA) was used at 10 µg/mL while the 8-OHdG antibody (Trevigen, Maryland, USA) was used at 2.5 µg/mL. In apoptotic assays, skin sections were subjected to routine hematoxylin and eosin staining for visualization of sunburn cells. The stained sections were examined under a Zeiss-Axioscan light microscope (Oberkochen, Germany) at 20 × magnification, and the number of sunburn cells per linear millimetre of skin section recorded as previously described^66^. Three areas of each section were analysed.

### Ultraviolet radiation–human studies

Studies on human tissues were approved by the Human Research Ethics Committee (University of Sydney, reference number 2015-063) and were conducted according to the Helsinki Declaration. All subjects, or legal guardians, gave written informed consent.

Human skin explant samples were obtained from two donors at the time of elective surgery and cut into pieces approximating 5 mm x 5 mm in area for placement into 96 well plates in buffered saline (PBS; pH 7.2) containing 5 mM D-glucose. Solar simulated UV-irradiation (ssUV) was provided by an Oriel 1000W xenon-arc lamp (Stratford, CT, USA). Energy level of irradiation was a mixture of 400 mJ/cm^2^ UVB and 3600 mJ/cm^2^ UVA measured by OL754 radiometer (Optronics Laboratories Inc., Orlando, FL) which equated to approximately 4 min irradiation of the Australian sun at noon in October in Sydney, Australia^68^. Non-irradiated control cells were simultaneously processed with irradiated cells but protected from ssUV. Skin explants in triplicate were irradiated and immediately afterwards exposed to either vehicle, 1,25(OH)_2_D_3_ or IK14800. Three hours after ssUV the explants were fixed and stained for analysis of CPD, 8-OHdG or sunburn cells.

Primary human keratinocyte cultures were established from skin biopsies obtained from three independent donors as per the method previously described^114^. Keratinocyte passages 1–5 were used in all experiments and the cells were plated on poly-D-lysine coated coverslips in 96 well plates and irradiated with ssUV. 1,25-dihydroxyvitamin D3 (1,25(OH)_2_D_3_) (Cayman Chemical, MI, USA) was solubilized in 100% spectroscopic ethanol (Merck, Darmstadt, Germany) and the concentration determined by spectroscopy (NanoDrop 2000, Thermo Fisher Scientific, MA, USA). Peptide IK14800 was solubilised in PBS. The vehicle control was 0.1% (v/v) spectroscopic ethanol and 0.1% (v/v) PBS. For all experiments 1,25(OH)_2_D_3_ and IK14800 were added immediately after ssUV. Three hours later the cells were fixed and stained for quantitation of CPD formation by image analysis^114^.

### MMP-1 (collagenase-1) expression

Human skin explants were prepared according to the protocol described by Song and colleagues^115^ apart from some minor changes. Skin was removed from two donors at elective surgery and cut into pieces for placement into one well of 96 well plates and skin explants in triplicate were used for each treatment condition. Skin tissues were subjected to UV-radiation from the Oriel 1000W xenon-arc lamp described above and then treated immediately afterwards with either IK14800 (concentrations in the range of 50-500 µM) or 1,25(OH)_2_D_3_ as positive control for 24 h. Explants were then fixed, paraffin-embedded and sectioned before being deparaffinised and rehydrated for staining. Citrate buffer was used for antigen retrieval and the sections were then stained for MMP-1 using a polyclonal antibody from Santa Cruz (which is now discontinued, i.e., Cat. No. sc-30074; rabbit polyclonal; clone H-72). Staining with an isotype control antibody (without primary antibody) was included in the protocol and Immunohistochemistry analysed using the Metamorph image analysis program^70^.

### HaCaT cell UVB irradiation and apoptosis study

The HaCaT cell line was obtained from the American Type Culture Collection resource centre and cultured in RPMI 1640 medium (Life Technologies, Thermo Fisher) supplemented with 5% (v/v) FBS (Sigma) and 1% (v/v) Penicillin–Streptomycin-Glutamine (Life Technologies, Thermo Fisher). Cell viability of adherent cells exposed to peptide was assessed after 24 h by means of CCK-8 reagent added for one hour followed by reading culture plates at 450 nm using a CLARIOstar plate reader (BMG LabTech, Cary, USA). Results were expressed as the mean ± SEM for 3 replicates performed in triplicate and were calculated as a percentage of the untreated controls with the viability of the latter expressed as 100%.

The UVB source was a UV cabinet (Wayne Electronics, Sydney, Australia) housed 6 UV fluorescent lamps: 3 UVA Cosmolux 15,504 40 W Sunlamps (Cosmedico, Stuttgart, Germany) and 3 UVB Phillips Ultraviolet 8 TL20 W/01 RS lamps (Phillips, Eindhoven, Holland). The variation in the output (mW/cm^2^) of the UVB lamps was measured using a UVB detector attached to an IL-1400A Photometer (International Light, Newburyport, USA). Adherent HaCaT cells were exposed to IK14800 (10 µM) for 24 h followed by removal of medium and washing the cell monolayer with PBS. Following addition of 0.5 mL PBS the cells were then sham- or UVB-irradiated (2 kJ/m^2^) after which the PBS was replaced with 1 mL tissue culture medium. At 16 h post-irradiation the cells were detached and then resuspended and stained with the Annexin V-FITC Apoptosis Staining/Detection Kit (containing Annexin V and Propidium Iodide) (Abcam ab14085) as per the manufacturer’s instructions. Cell analysis was performed with the BD-FACSCanto flow cytometer using the FACS Diva software. Ten thousand events were recorded for each run. Detection was performed using a detection filter 488/530 for Annexin-FITC, and 488/610 for PI. Each experiment was performed in duplicate and repeated twice, and the results expressed as mean ± SD.

### Statistical analyses

For flow cytometry and ELISA experiments, data from IK14800 and vehicle groups were analysed using parametric statistical procedures. Data within groups to be compared were assumed to be normally distributed and to satisfy the homogeneity of variance criterion. Data comparisons between peptides and vehicle control were made using repeated measures (RM) two-way ANOVA with Dunnett’s post-test and comparisons between IK14800 and control peptide (IK14000) were made using two-way ANOVA with Sidak’s post-test comparing matched concentrations of IK14800 and IK14000. Data from flow cytometry/ELISA assays were analysed using GraphPad Prism (version 8.4.2)^116^. Data from the B16F10 murine melanoma study were analysed using one-way ANOVA post-hoc comparison on a GraphPad Prism program (version 8.3.1)^116^. Statistical analyses of data obtained from the solar radiation murine and human tissue experiments were performed using ANOVA with Tukey’s post-test on a GraphPad Prism program (version 8.1.1)^116^. All three GraphPad Prism programs were performed on a Windows Operating System.

## Supplementary Information

Supplementary Figures.

## Data Availability

Dot plot/gating strategies for flow cytometry studies are available in the Supplementary Information provided. All remaining raw datasets used and/or analysed during the current study are available from the corresponding author on reasonable request.
