# Supplementary material for: An immunomodulating peptide to counteract solar radiation-induced immunosuppression and DNA damage
Source: Sci Rep. 2023 Jul 20;13:11702. doi: 10.1038/s41598-023-38890-4 (PMC10359417; doi:10.1038/s41598-023-38890-4)
Supplement: Supplementary file 1 — Supplementary Figures. [file 41598_2023_38890_MOESM1_ESM.pdf]

An immunomodulating peptide to counteract solar radiation-induced immunosuppression and DNA damage

Michael Agrez, Mark Stephen Rybchyn, Warusavithana Gunawardena Manori De Silva, Rebecca Sara Mason, Christopher Chandler, Terrence J. Piva, Kristofer Thurecht, Nicholas Fletcher, Feifei Liu, Gayathri Subramaniam, Christopher B. Howard, Benjamin Blyth, Stephen Parker, Darryl Turner, Justyna Rzepecka, Gavin Knox, Anastasia Nika, Andrew Hall, Hayley Gooding and Laura Gallagher

Supplementary information online flow cytometry data

Figure S1: Refers to manuscript **Figure 1a:** Viability (CD3 72 hrs)

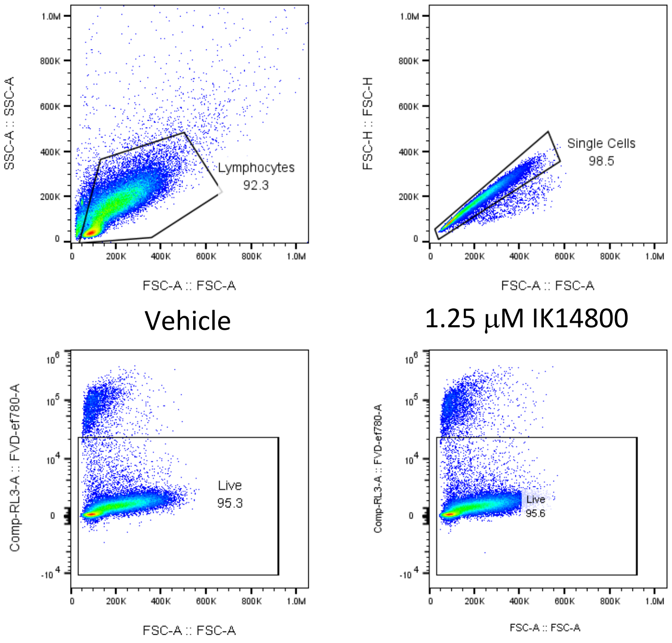

Figure S2: Refers to manuscript **Figures 1g and 1h:** CD25 % in CD4+ and CD8+ (PBMC 24 hrs)

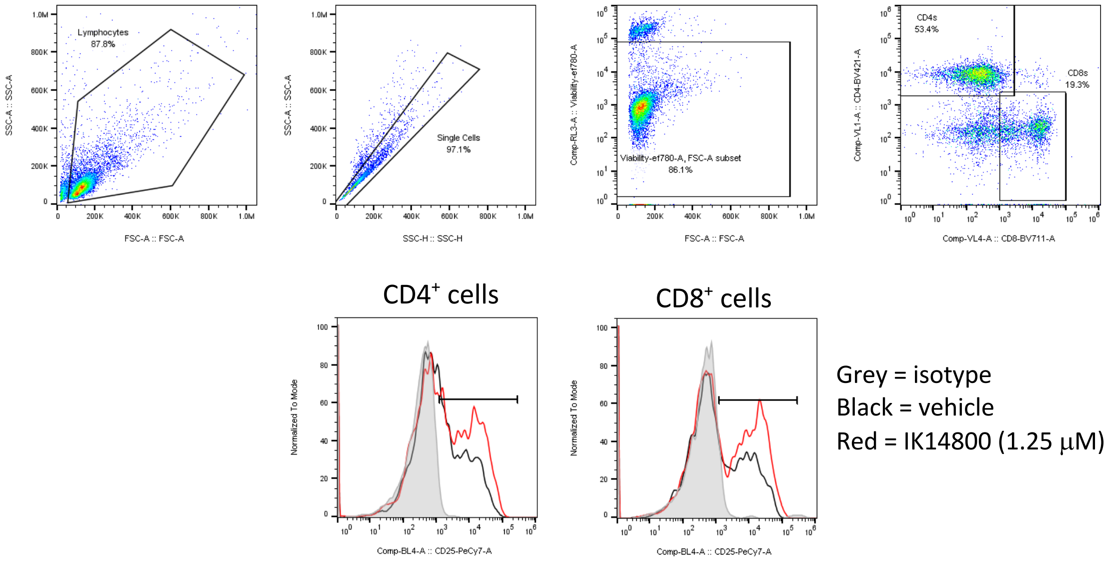

Figure S3: Refers to manuscript **Figures 1i and 1j:** CD25 % in CD4+ and CD8+ (PBMC 24 hrs)

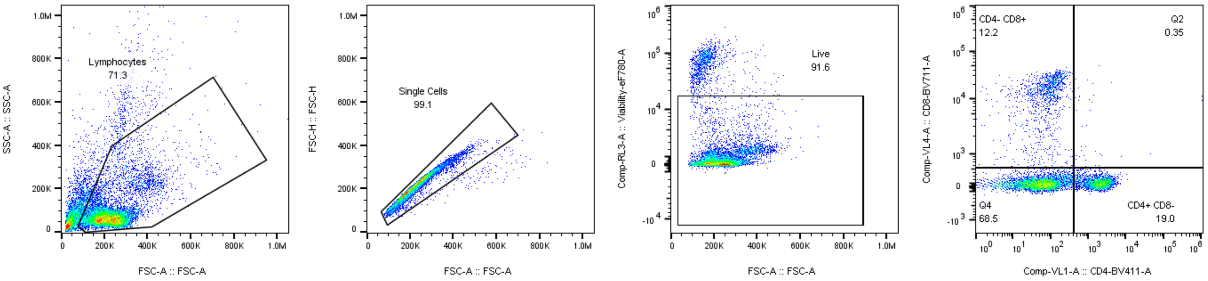

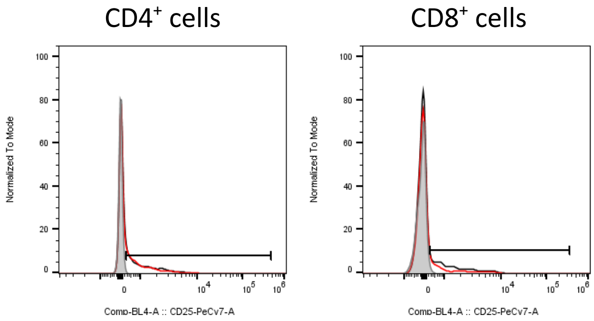

Grey = isotype
Black = vehicle
Red = IK00900 (1.25 μM)

Figure S4: Refers to manuscript **Figure 1k:** Ki67 CD4+ MFI (PBMC 72 hrs)

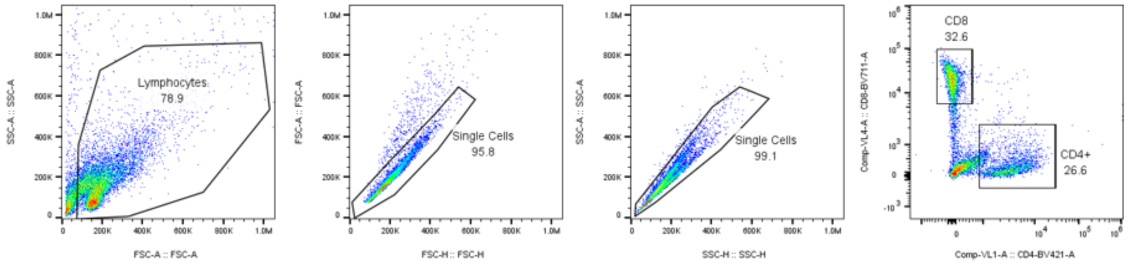

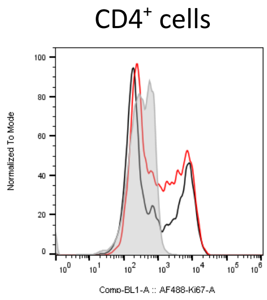

Grey = Isotype
Black = vehicle
Red = IK14800 (1.25 μM)

Figure S5: Refers to manuscript **Figure 1l:** Foxp3+ % in CD4+ CD25+ CD127^{low} (PBMC 48 hrs)

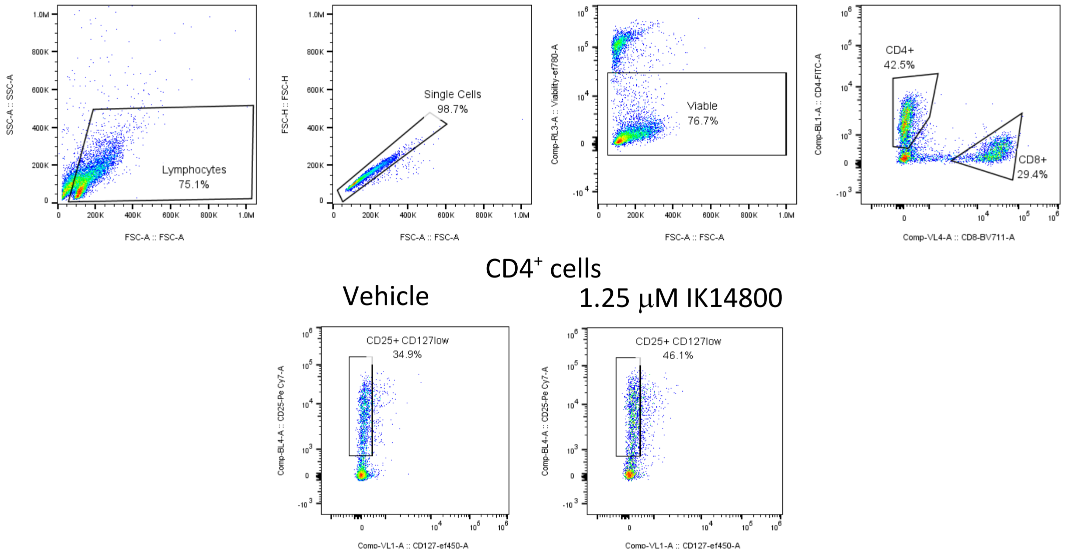

Figure S6: Refers to manuscript **Figures 1m and 1n:** CD28 MFI (CD4 72 hrs)

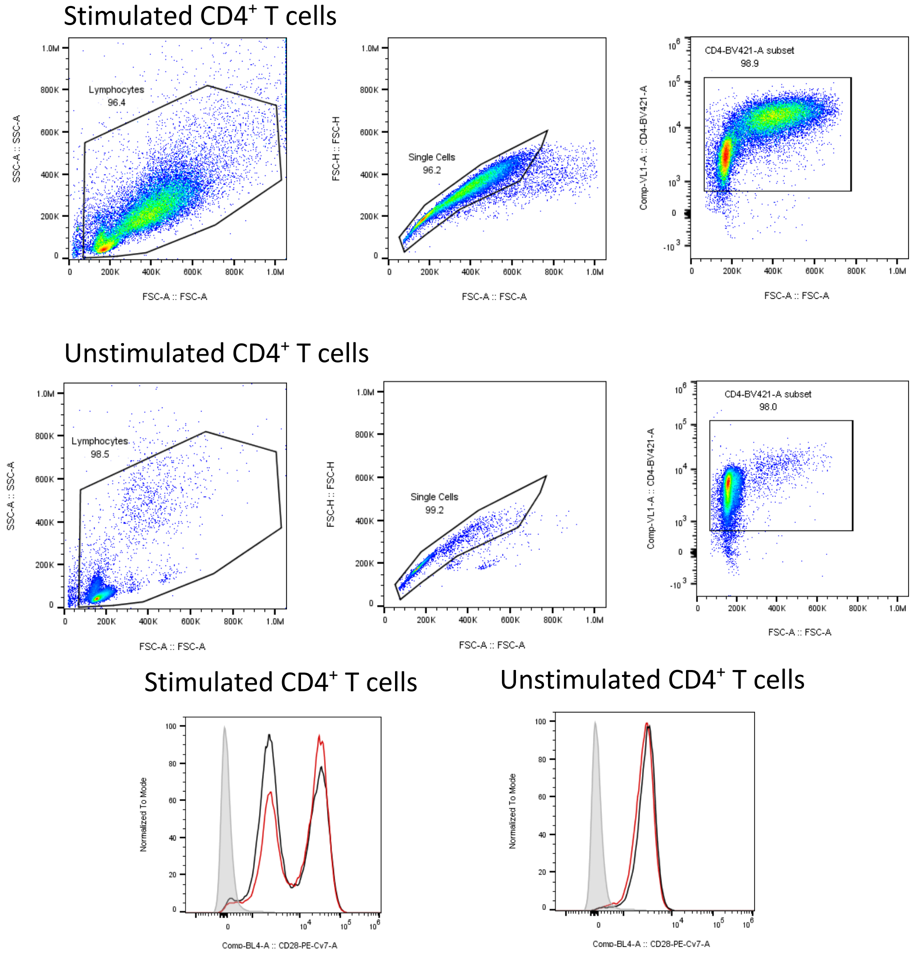

Figure S7: Refers to manuscript **Figure 1o:** CD28 MFI in CD4+ young and aged donors (CD4 72 hrs)

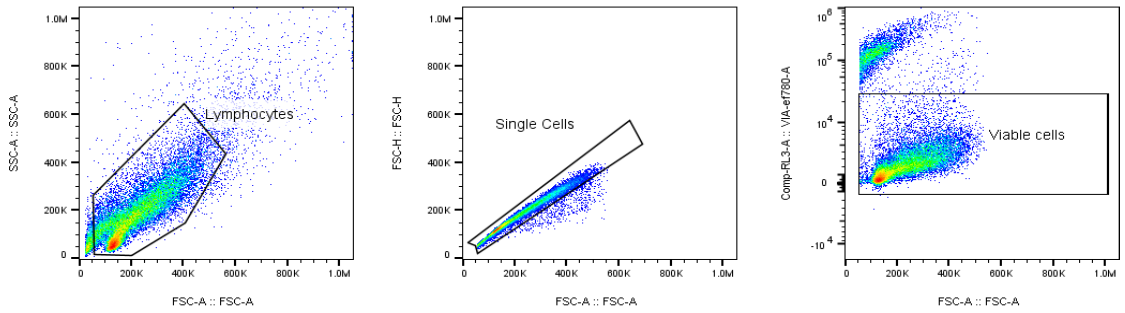

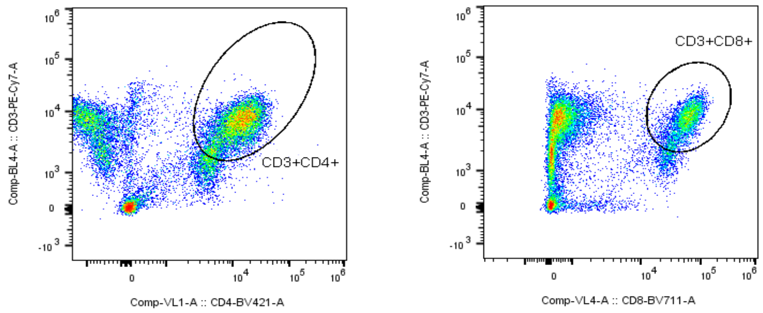

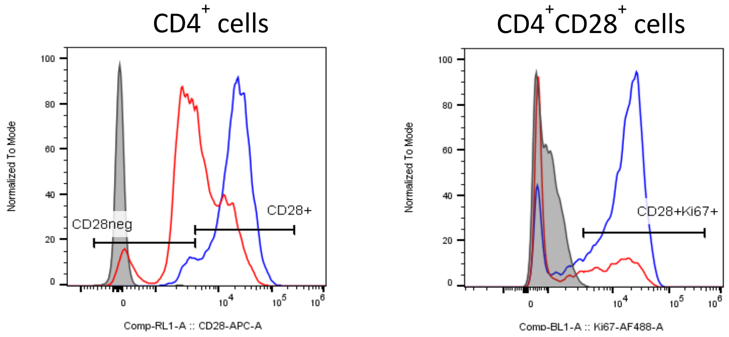

Grey = FMO
Blue = young
Red = aged

Figure S8: Refers to manuscript **Figure 1p:** CD28 MFI in CD4+ aged donors (CD4 72 hrs)

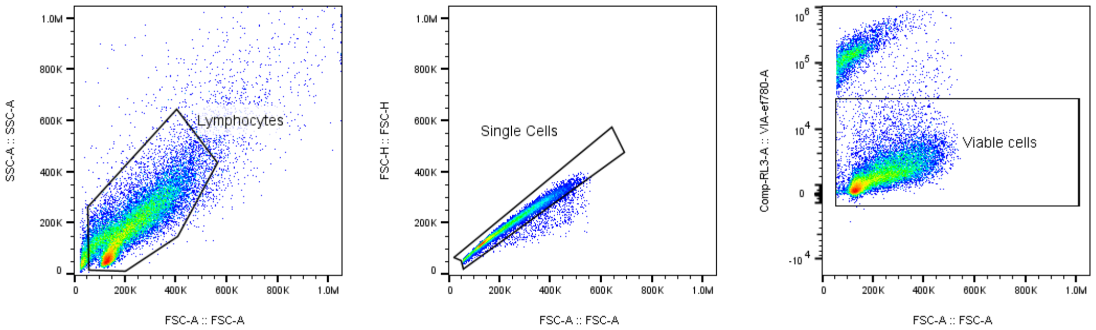

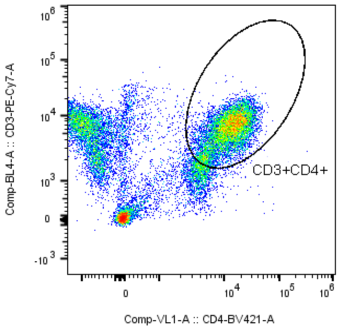

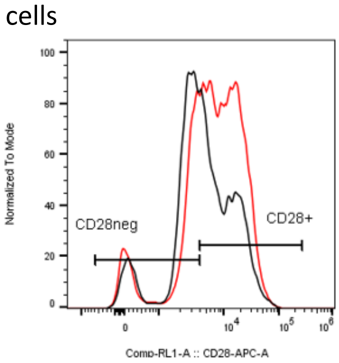

Black = vehicle
Red = IK14800 (1.25 μM)

Figure S9: Refers to manuscript **Figures 2a and 2b:** Intracellular IFN-γ % in CD4+ CD8+ (PBMC 24 hrs)

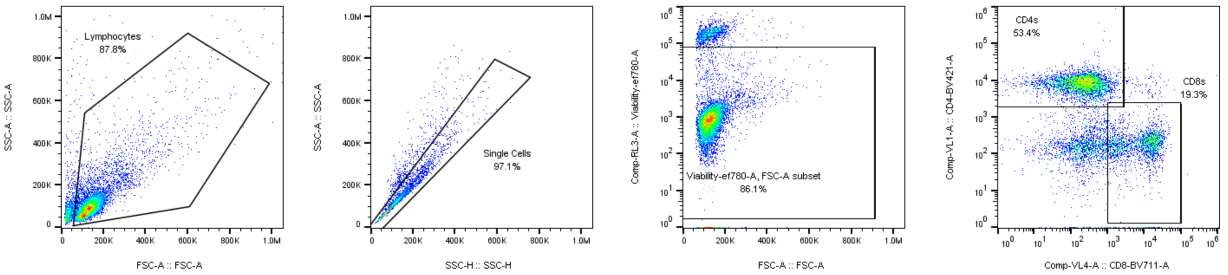

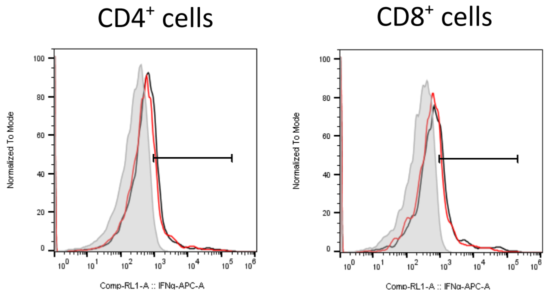

Grey = isotype
Black = vehicle
Red = IK14800 (1.25 μM)

Figure S10: Refers to manuscript **Figure 2f:** IL-18Rα MFI in CD4+ (CD4 24 hrs)

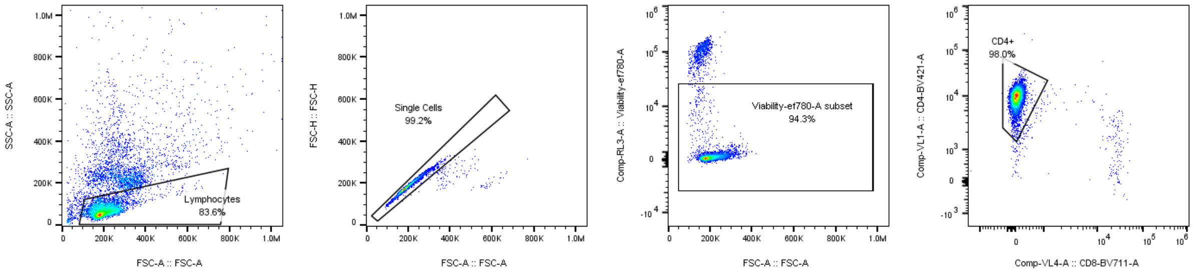

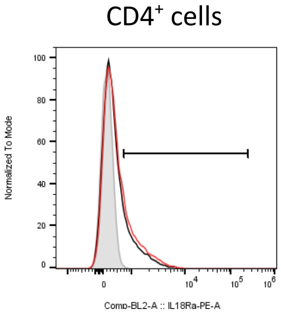

Grey = isotype
Black = vehicle
Red = IK14800 (1.25 μM)

Figure S11: Refers to manuscript **Figure 2g:** IL-18Rα MFI in CD8+ (CD4 24 hrs)

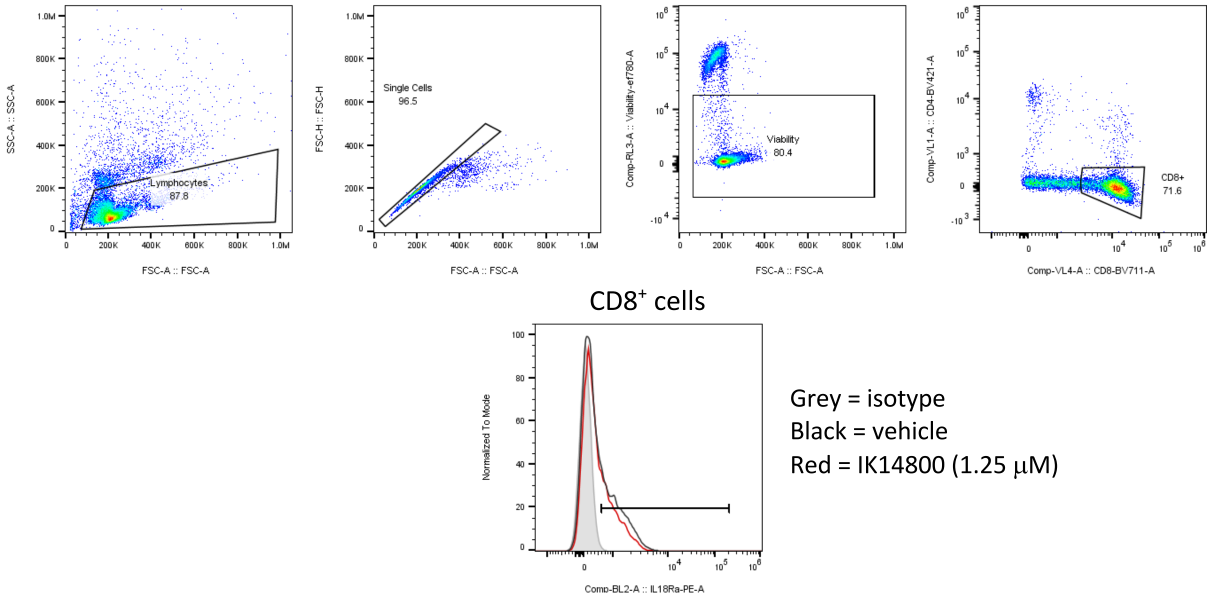

Figure S12: Refers to manuscript **Figures 3a and 3b:** Viability and CD14± DCs iMoDC (72 hrs)

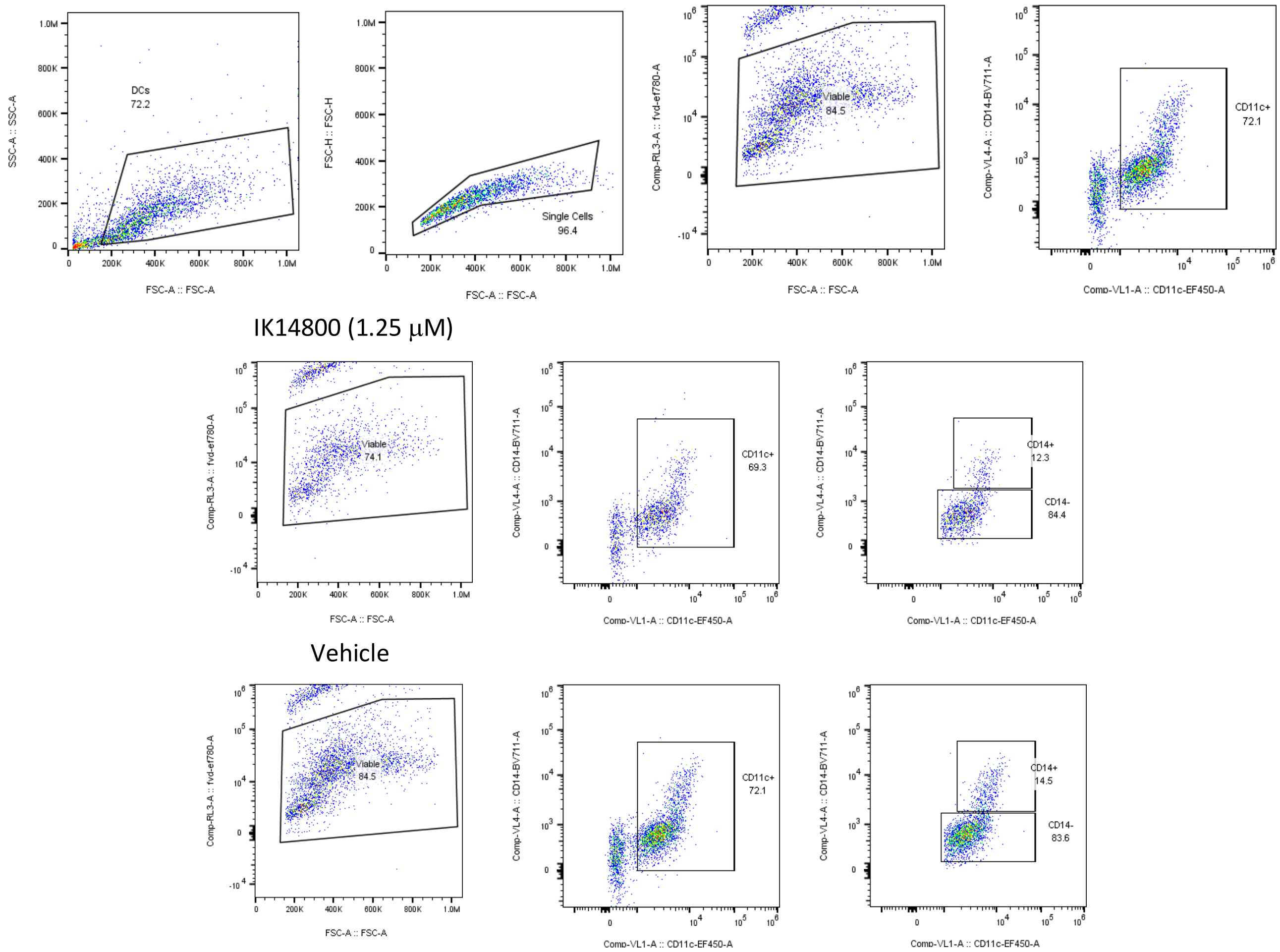

Figure S13: Refers to manuscript **Figures 4d to 4g:** CD25 MFI and % in exhausted CD4+ (CD4 72 hrs)

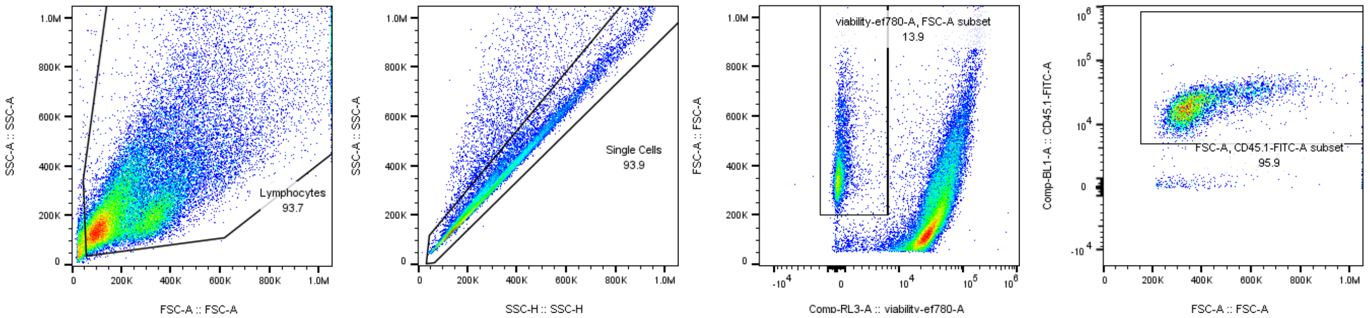

IK14800 1.25 µM (red) vs Isotype (grey)

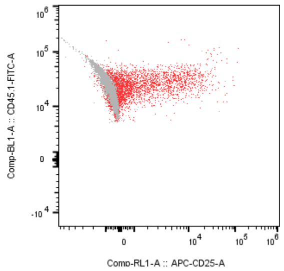

IK14000 1.25 µM (red) vs Isotype (grey)

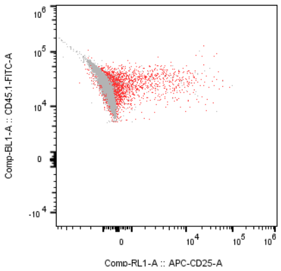

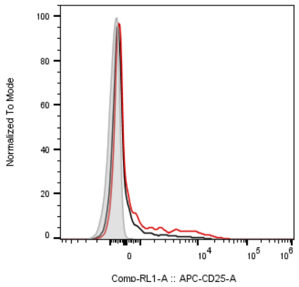

IK14800 1.25 µM (red) vs Isotype (grey) vs IK14000 (black)

Figure S14: Refers to manuscript **Figures 6f and 6g:** Early/late apoptosis (HaCaTs: -/+ UVB)

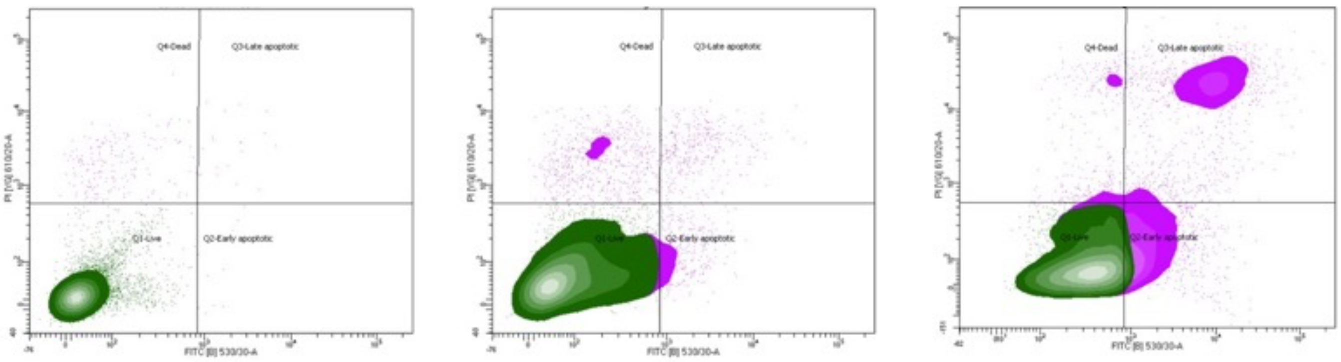

minus UVB minus IK14800 | minus UVB +IK14800 | +UVB +IK14800
